# Abscisic acid‐induced cytoplasmic translocation of constitutive photomorphogenic 1 enhances reactive oxygen species accumulation through the HY5‐ABI5 pathway to modulate seed germination

**DOI:** 10.1111/pce.14298

**Published:** 2022-03-10

**Authors:** Qing‐Bin Chen, Wen‐Jing Wang, Yue Zhang, Qi‐Di Zhan, Kang Liu, José Ramón Botella, Ling Bai, Chun‐Peng Song

**Affiliations:** ^1^ State Key Laboratory of Crop Stress Adaptation and Improvement, School of Life Sciences Henan University Kaifeng China; ^2^ Department of Biology and Food Science Shangqiu Normal University Shangqiu China; ^3^ Plant Genetic Engineering Laboratory, School of Agriculture and Food Sciences The University of Queensland Brisbane Queensland Australia

**Keywords:** ABA, COP1, hormones, HY5‐ABI5 pathway, oxidative stress, seed germination

## Abstract

Seed germination is a physiological process regulated by multiple factors. Abscisic acid (ABA) can inhibit seed germination to improve seedling survival under conditions of abiotic stress, and this process is often regulated by light signals. Constitutive photomorphogenic 1 (COP1) is an upstream core repressor of light signals and is involved in several ABA responses. Here, we demonstrate that COP1 is a negative regulator of the ABA‐mediated inhibition of seed germination. Disruption of COP1 enhanced *Arabidopsis* seed sensitivity to ABA and increased reactive oxygen species (ROS) levels. In seeds, ABA induced the translocation of COP1 to the cytoplasm, resulting in enhanced ABA‐induced ROS levels. Genetic evidence indicated that HY5 and ABI5 act downstream of COP1 in the ABA‐mediated inhibition of seed germination. ABA‐induced COP1 cytoplasmic localization increased HY5 and ABI5 protein levels in the nucleus, leading to increased expression of ABI5 target genes and ROS levels in seeds. Together, our results reveal that ABA‐induced cytoplasmic translocation of COP1 activates the HY5‐ABI5 pathway to promote the expression of ABA‐responsive genes and the accumulation of ROS during ABA‐mediated inhibition of seed germination. These findings enhance the role of COP1 in the ABA signal transduction pathway.

## 1 INTRODUCTION

Seed germination needs to be tightly regulated in the life cycle of flowering plants, as germination in unfavourable conditions can lead to seedling death. Given its critical importance for survival, seeds can sense a variety of internal signals and environmental cues, and the interplay between all factors regulates the process of germination (Finch‐Savage & Leubner‐Metzger, 2006; Penfield, 2017; Yang et al., 2020). Knowledge of the mechanisms by which these factors are integrated into a regulation network remains fragmented.

The phytohormone abscisic acid (ABA) has a critical inhibitory role in seed germination, and the molecular mechanisms of this inhibition have been extensively studied. In *Arabidopsis*, ABA is sensed by pyrabactin resistance (PYR)/PYR1‐like (PYL)/regulatory components of ABA receptor (RCAR), resulting in the dissociation of protein phosphatase 2C (PP2C) from SNF1‐related protein kinase 2 (SnRK2) Kinase. Free SnRK2 Kinase then phosphorylates downstream factors to activate the ABA signal transduction pathway (Ma et al., 2018). Many components of the ABA signalling pathway can directly affect seed germination. ABA‐biosynthesis mutants (*nced3*, *nced6*, *aba2*) and ABA signalling repressor ABI1 mutants (*abi1‐1*, *abi1‐2*) germinate faster than the wild type (WT) and show decreased seed dormancy (Shu et al., 2013). Moreover, transcription factors such as ABA insensitive 3 (ABI3), ABA insensitive 4 (ABI4) and ABA insensitive 5 (ABI5) play important roles in ABA inhibition of seed germination, by positively regulating the expression of ABA‐responsive genes, such as *RD29A* and *RD29B* (Skubacz et al., 2016). In addition, ABI5 also acts as a central integrator between ABA and other signalling pathways (Yadukrishnan & Datta, 2021).

In addition to ABA, light, an important exogenous signal, regulates seed germination by affecting the ABA/GA balance in seeds. Recent studies have shown that components of the light signal transduction pathway mediate ABA‐mediated inhibition of seed germination (Seo et al., 2009; Yadukrishnan & Datta, 2021). Under ABA treatment, phytochrome interacting factor 4 (PIF4) and phytochrome interacting factor 5 (PIF5) interact with the ABA receptors PYR1‐like 8 (PYL8) and PYR1‐like 9 (PYL9) to promote PIF4 and PIF5 accumulation in darkness and enhance PIF4 and PIF5 binding to the ABI5 promoter (Qi et al., 2020). Moreover, the transcription factors elongated hypocotyl 5 (HY5), HY5‐homologue (HYH) and B‐box domain protein 21 (BBX21) involved in photomorphogenesis can directly bind to the ABI5 promoter to regulate the expression of ABI5 (Xu et al., 2014). ABA promotes binding of HY5 to the ABI5 promoter resulting in enhanced ABI5 expression. The HY5‐ABI5 pathway mediates inhibition of seed germination during abiotic stresses, such as salt stress, high temperature, and so forth (Chen et al., 2019; Wang et al., 2021; Yu et al., 2016). BBX21 interacts with HY5 interfering with the HY5‐mediated induction of ABI5 transcription, while BBX21 directly interacts with ABI5 weakening ABI5 binding to its own promoter (Chen et al., 2008; Kang et al., 2018; Xu et al., 2014). Although some downstream components of the light signalling pathway have been shown to regulate ABA inhibition of seed germination, little is known about the involvement of upstream light signalling components.

Constitutive photomorphogenic 1 (COP1) is an upstream core repressor in the light signal transduction pathway and exert its role by ubiquitinating transcription factors involved in photomorphogenesis to promote skotomorphogenesis (Han et al., 2020). In plants, COP1 binds SPA (suppressor of PhyA‐105) to form a COP1‐SPAS protein complex, regulating many physiological and biochemical processes. COP1's nucleocytoplasmic partitioning plays an important role in controlling its function although the molecular mechanisms controlling such partitioning are still unclear (Balcerowicz et al., 2017; Podolec & Ulm, 2018). The role of COP1 is not restricted to light signalling, and it has also been implicated in the physiological response to phytohormones, clock regulation, temperature perception, biotic stress, and so forth (Jeong et al., 2010; Martinez et al., 2018; Wang et al., 2019; Xu, Zhu, et al., 2016). Several roles for COP1 in phytohormone signalling have gradually emerged, although its involvement in ABA signalling is relatively obscure. Absence of COP1 reduces stomatal sensitivity to ABA, weakens ABA‐induced microtubule depolymerization in guard cells, and reduces the activity of SLAC1 (slow anion channel‐associated1) in guard cells (Khanna et al., 2014). Recent studies have shown that COP1 regulates ABA‐induced stomatal response by degrading the negative ABA regulators protein phosphatase type 2Cs (PP2Cs) and activating ABA downstream signalling pathways (Chen et al., 2021). Aside from the two mentioned examples, the involvement of COP1 in ABA‐mediated responses is unknown.

Moreover, reactive oxygen species (ROS) act downstream of the ABA signalling pathway to mediate ABA inhibited seed germination (Bailly, 2019; Oracz & Karpinski, 2016; Postiglione & Muday, 2020). ROS include singlet oxygen (^1^O_2_), superoxide anion (O_2_^−^), hydrogen peroxide (H_2_O_2_) and hydroxyl radical (OH^−^), with H_2_O_2_ playing an important signalling role (Bailly, 2019). Low ROS levels in the seed enhance ABA catabolism and gibberellin (GA) biosynthesis, promoting germination (Liu et al., 2010). In contrast, uncontrolled accumulation of ROS under stress conditions can damage the cell membrane and small molecules in the cell (such as nucleic acids, lipids, protein, etc.), leading to the delay or inhibition of seed germination (Mittler, 2002, 2006). The ABA‐induced increase in ROS levels in seeds is closely linked to the enhanced expression of the NADPH oxidases *RbohD* and *RbohF*, which are key components in the production of ROS (Choudhury et al., 2017; Torres & Dangl, 2005). However, the regulatory mechanisms controlling ABA‐induced ROS accumulation during seed germination remain elusive, and the roles of light signalling components in this process are largely unknown.

In this article, we demonstrate that COP1 negatively regulates ABA‐mediated inhibition of seed germination. We found that disruption of COP1 enhanced seed sensitivity to ABA. In seeds, ABA‐induced COP1 accumulation in the cytoplasm. In turn, ABA‐induced COP1 cytoplasmic localization increased HY5 and ABI5 protein levels in the nucleus, leading to increased expression of ABI5 target genes and raised ROS levels in seeds.

## 2 MATERIALS AND METHODS

### 2.1 Plant materials and growth

*cop1‐4*, *cop1‐6* and *GUS‐COP1* Arabidopsis lines used in this study had Columbia ecotype background, *hy5‐ks50* and *abi5‐1* had Wassilewskija ecotype background. *CFP‐COP1*, *COP1* ^*nu*^ *−1*, *COP1* ^*nu*^ *−2*, *COP1* ^*cty*^ *−1*, *COP1* ^*cyt*^ *−2* transgenic lines were generated by 35S promoter driving the expression of *COP1*, *COP1* ^*nu*^ or *COP1* ^*cyt*^ in the *cop1‐4* background. Double *cop1‐4/hy5‐ks50*, *cop1‐4/abi5‐1*, *COP1* ^*nu*^ *−1/abi5‐1*, and *COP1* ^*cyt*^ *−1/abi5‐1* mutants were generated by genetic crossing. The primers used for identification of the mutations in the progeny of crosses are listed in Table S1. Seeds were vernalized at 4°C and grown on MS medium (M519, Murashige & Skoog basal medium with vitamins) with pH at 5.6 for 7 days before transplanting to soil, and grown in a greenhouse with 12‐h light/12‐h dark cycle (100 μmol photons m^−2^ sec^−1^).

### 2.2 Seed germination

After surface disinfection, *Arabidopsis thaliana* seeds were sown on MS medium containing 0, 0.1 or 0.5 μM ABA, vernalized at 4°C for 2 days, and placed in an incubator at 22°C for germination experiments. The time at which seeds were placed in the culture room was considered as 0 h, germination was determined by protrusion of the radicle from the seed coat, and recorded every 12 h. More than 50 seeds per genotype were used for each experimental condition, and every experiment repeated three times. Germination rate was calculated as the proportion of germinated seeds from the total in each experiment. Seeds were germinated in an incubator at 22°C with 12‐h light/12‐h dark cycle (100 μmol photons m^−2^ sec^−1^).

### 2.3 Nitroblue tetrazolium (NBT) staining assay

NBT staining assay of germinated seeds was performed as described by Wang et al. (2020) with slight modifications. Five milligrams of NBT were dissolved in 6 ml of 20 mM potassium phosphate buffer (pH 6.7) containing 0.1 M NaCl. Thirty seeds were germinated for 48 h on MS medium containing 0 or 0.5 μM ABA and incubated in 1 ml NBT staining solution for 15 min. After staining, the seeds were first incubated in acidic methanol solution (5 ml methanol, 1 ml HCl and 4 ml ddH_2_O) at 57°C for 15 min, then quickly transferred to an alkaline solution (3.5 g NaOH, 30 ml ethanol and 20 ml ddH_2_O) for 15 min at room temperature. Finally, seeds were incubated in a series of ethanol concentrations to remove colour. Seeds were photographed with a microscope fitted with a 10x lens, and adobe photoshop was used to analyse the dye density which was measured as pixel counts in the seeds' root images.

### 2.4 ROS determination

The hydroxylamine oxidation method was used to measure O_2_^−^ content. Briefly, 50 mg of germinated seeds were ground in liquid nitrogen in a small mortar. After grinding, 250 μl 50 mM phosphate buffer (pH 7.8) was added and the mix centrifuged at 4°C for 30 min at ×12 000*g*. Supernatant (100 μl) was added to 50 μl 50 mM phosphate buffer (pH 7.8) and 50 μl 10 mM hydroxylamine hydrochloride and incubated at room temperature for 30 min. After incubation, 100 μl 17 mM 4‐Aminobenezenesulfonic acid and 100 μl 7 mM α‐Naphthylamine were added, and further incubated at room temperature for 15 min. The absorption value of the clarified supernatant from the reaction solution was detected by a microplate reader at 530 nm, and the content of nitrite in the reaction solution calculated using standard curve. Finally, the O_2_^−^ content was calculated by the molecular ratio of nitrite and O_2_^−^ in the hydroxylamine oxidation reaction.

Titanium Sulphate oxidation was used for the determination of H_2_O_2_ content in seeds (Nakayama et al., 1983). Briefly, 100 mg germinated seeds were grinded with liquid nitrogen. After grinding, 300 μl acetone was added and the mix centrifuged for 10 min at ×12 000*g*, 4°C. Supernatant (100 μl) was added to 10 μl 5% (w/v) titanium sulphate and 20 μl concentrated ammonia and centrifuged at ×5000*g* for 10 min. The supernatant was discarded and the pellet dissolved in 500 μl 2 M H_2_SO_4_. The H_2_O_2_ produced by this reaction was detected by light absorption at 410 nm and quantified using a standard curve.

### 2.5 Measurement of NADPH oxidase and antioxidant enzyme activities

NADPH oxidase activity was analysed as described by Rojas et al. (2012), with some modifications. Germinated seeds (1 g) were ground with liquid nitrogen, and homogenized in homogenizing buffer (15 mM Tris‐HCl, pH 7.8, 1 mM EDTA, 4 mM DTT, 1 mM PMSF, 0.6% [w/v] PVP, 0.25 M sucrose). Initial residue was removed by centrifugation at ×12 000*g* for 30 min at 4°C, and fractions obtained by centrifugation at ×203 000*g* for 60 min at 4°C. The pellet was re‐suspended in dilution buffer (10 mM Tris‐HCl, pH 7.4, 0.25 M sucrose, 1 mM DTT). Twenty micrograms of membrane protein was added into NADPH oxidase activity reaction solution (50 mM Tris‐HCl, pH 7.4, 0.5 mM XTT, 100 mM NADPH) and incubated at 30°C for 5 min. Light absorption values at 470 nm were recorded and the rate of O_2_^−^ generation calculated using an extinction coefficient of 2.16 × 10^4 ^m^−1^ cm^−1^ (Jiang & Zhang, 2002).

SOD, CAT, POD and APX activities in this study were quantified using commercial kits from Solarbio Life Science (Beijing, CPRC) following manufacturers' instructions. The enzyme activity values correspond to the means of three biological replicates ±SD.

### 2.6 Subcellular localization of GUS‐COP1

Subcellular localization of GUS‐COP1 was performed as described by Yu et al. (2016) with minor modifications. Transgenic *GUS‐COP1* seeds germinated on MS medium containing 0 or 0.5 μM ABA were fixed with 90% cold acetone for 30 min before staining overnight in a GUS staining solution (0.5 mg/ml 5‐bromo‐4‐chloro‐3‐indolyl‐β‐d‐glucuronic acid, 5 mM ferricyanide and ferrocyanide, 10 mM EDTA, and 0.1% [v/v] Tween 20 in 100 mM sodium phosphate, pH 7.0) at 37°C. After staining, seeds were heated at 60°C for 5 min in 70% ethanol and transferred to a dissociation solution (15% [v/v] HCl and 95% [v/v] ethanol, 1:1) at 60°C for 3–5 min. After rinsing with ddH_2_O three times, the seeds were stained in 0.5 μg/ml DAPI buffer. DAPI stained seeds were rinsed with ddH_2_O three times were placed on microscope slides and cover slides gently tapped with tweezers to disperse the DAPI stained seeds into single cells. A fluorescence microscope was used to photograph the results.

### 2.7 Cell fractionation assay

Nuclear and cytoplasmic proteins were extracted as described by Wang et al. (2018). Briefly, 0.5 g of germinated seeds were ground in liquid nitrogen and mixed with 2 ml of fractionation lysis solution (20 mM HEPES, pH 7.5, 25% [v/v] glycerine, 2 mM EDTA, 2.5 mM MgCl_2_, 20 mM KCl, 250 mM sucrose, 5 mM DTT and the mixture of protease inhibitor [Roche]). The mix was filtered using a double Miracloth (Millipore) and centrifuged at ×1500*g* for 10 min at 4°C. At this point, precipitate and soluble fractions were separated and subjected to different treatments. The precipitate was washed several times with NRBT buffer (20 mM Tris‐HCl, pH 7.5, 25% [v/v] glycerine, 2.5 mM MgCl_2_ and 0.2% [v/v] Triton X‐100) until it became white (5–8 times) and resuspended in 400 μl NRB2 (20 mM Tris‐HCl, pH 7.5, 0.25 M sucrose, 10 mM MgCl2, 0.5% Triton X‐100 and 5 mM β‐mercaptoethanol, with the addition of protease inhibitor mixture). The suspension was carefully layered on top of 400 μl NRB3 (20 mM Tris‐HCl, pH 7.5, 1.7 M sucrose, 10 mM MgCl_2_, 0.5% [v/v] Triton X‐100 and 5 mM β‐mercaptoethanol, supplemented with protease inhibitor mixture) and centrifuged ×16 000*g* at 4°C for 45 min. The precipitate was collected as nuclear fraction. The supernatant from the initial low‐speed centrifugation (×1500*g*) was centrifuged at ×10 000*g* at 4°C for 10 min, and the precipitate collected as cytoplasmic fraction. Western blot analysis was performed using anti‐GFP antibody. Actin was used as an internal marker for cytoplasmic proteins and proliferating cell nuclear antigen (PCNA) was used as an internal marker for nuclear proteins. Three biological replicates were performed.

### 2.8 Quantitative Real‐Time PCR

An RNA purification kit (Tiangen) was used to extract RNA from germinating seeds, and DNA contamination removed by DNaseI (Invitrogen) treatment. cDNA was synthesized from 2 μg RNA using oligo dT primers (TaKaRa) and MMLV Reverse Transcriptase (Promega). Quantitative real‐time PCR was performed using the SYBR Taq Premix system (TaKaRa) on a LightCycler480 detection system (Roche) according to the manufacturer's instructions. The *Actin2* gene was used as an internal control for normalization purposes. Three biological replicates and three technical replicates were performed for each treatment. The primer sequences are listed in Table S1.

## 3 RESULTS

### 3.1 COP1 acts as a negative regulator of the ABA‐mediated inhibition of seed germination in *Arabidopsis*

**Figure 1:**
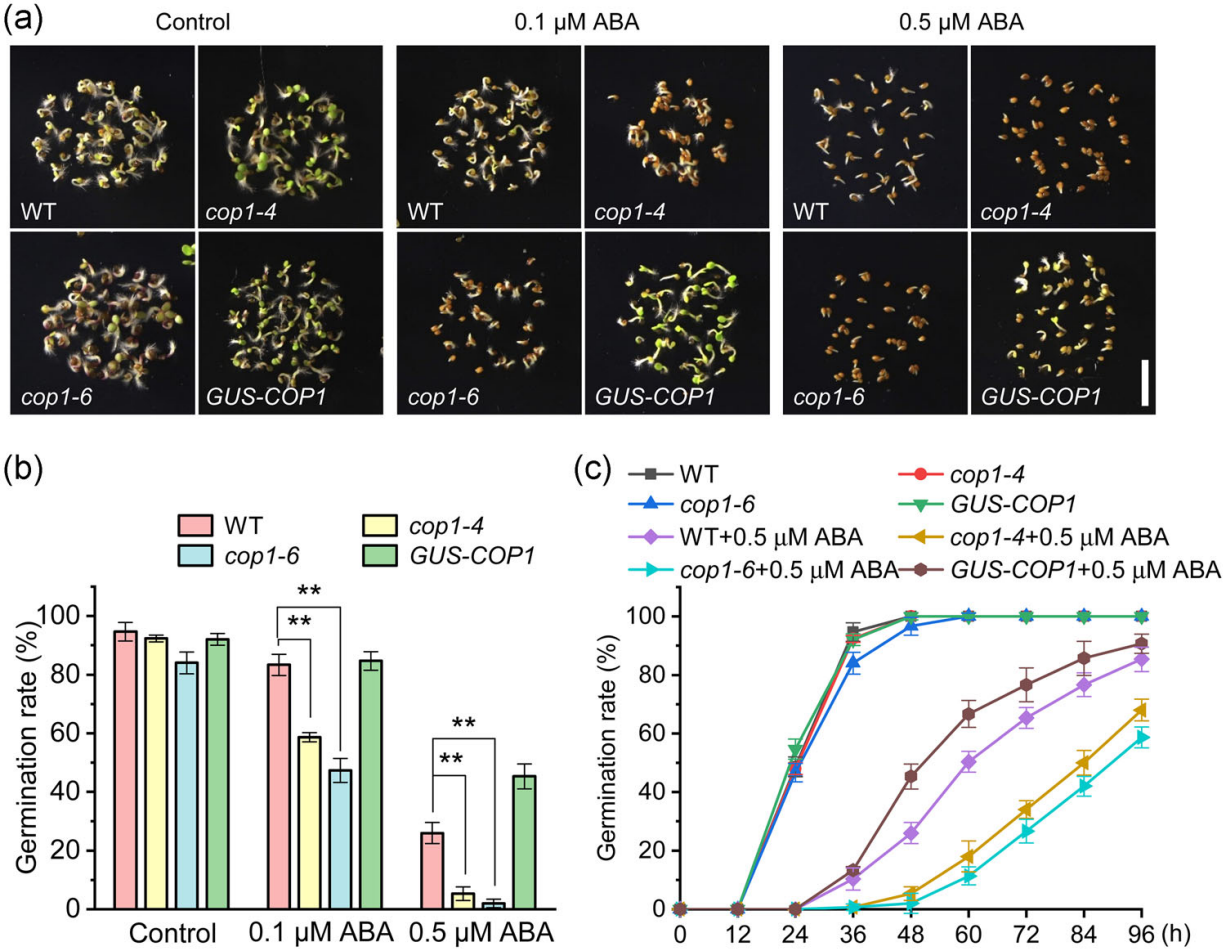
Mutations in *constitutive photomorphogenic 1* (*COP1*) increase abscisic acid (ABA)‐mediated inhibition of germination in *Arabidopsis*. (a) Germination phenotypes of wild type (WT), *cop1‐4* and *cop1‐6* mutants and the functional complementation line *GUS‐COP1*. Seeds were grown on media containing 0, 0.1 or 0.5 μM ABA for 48 h (scale = 0.5 cm). (b) Germination rates of all genotypes shown in (a). Data are means ± SD, *n* > 50 for three independent experiments. **p < 0.01; student's *t*‐test. (c) Germination rates of WT, *cop1‐4*, *cop1‐6* and *GUS‐COP1* seeds in media with or without ABA (0.5 μM). After vernalization, seeds were placed in an incubator and recorded as 0 h. Germination was measured every 12 h until reaching 96 h. Data are means ± SD, *n* > 50 for three independent experiments [Color figure can be viewed at wileyonlinelibrary.com]

In a previous study on the role of COP1 in stomatal movement (Chen et al., 2021), we obtained anecdotal evidence that seed germination under ABA treatment is different in COP1‐deficient mutants compared with WT. To follow on those observations, we performed germination assays in WT, two independent *cop1* mutants (*cop1‐4* and *cop1‐6*) and a previously characterized functional complementation line (*GUS‐COP1*) (Yu et al., 2016) in the absence or presence of ABA (Figure 1). In the absence of ABA all genotypes showed similar germination rates (Figure 1a,b), but the germination rates of *cop1‐4*, *cop1‐6* mutants were significantly lower than those of WT and *GUS‐COP1* under the two ABA concentrations assayed (0.1 μM and 0.5 μM ABA) (Figure 1a,b). Germination rates of *cop1‐4*, *cop1‐6* seeds in media containing 0.1 μM ABA (58.6% and 47.3%) were significantly lower than those of WT (83.3%) and *GUS‐COP1* (84.6%) seeds after 48 h (Figure 1a,b). Similarly, germination rates of *cop1‐4* and *cop1‐6* seeds sawn on media containing 0.5 μm ABA (5.3% and 2%) were also significantly lower than those of WT (26%) and *GUS‐COP1* (45.3%) (Figure 1a,b). In addition, we examined the germination dynamics of WT, *cop1‐4*, *cop1‐6* and *GUS‐COP1* seeds in the presence or absence of 0.5 μM ABA for 96 h. Differences in germination rates in 0.5 μM ABA between *cop1‐4* and *cop1‐6* lines and WT and *GUS‐COP1* lines was initially detected 36 h after sowing and remained for the rest of the time examined (Figure 1c, Figure S1). These results suggest that the absence of COP1 enhances the inhibitory effect of ABA on seed germination of *A. thaliana*.

### 3.2 COP1 regulates ABA‐induced ROS accumulation during seed germination

**Figure 2:**
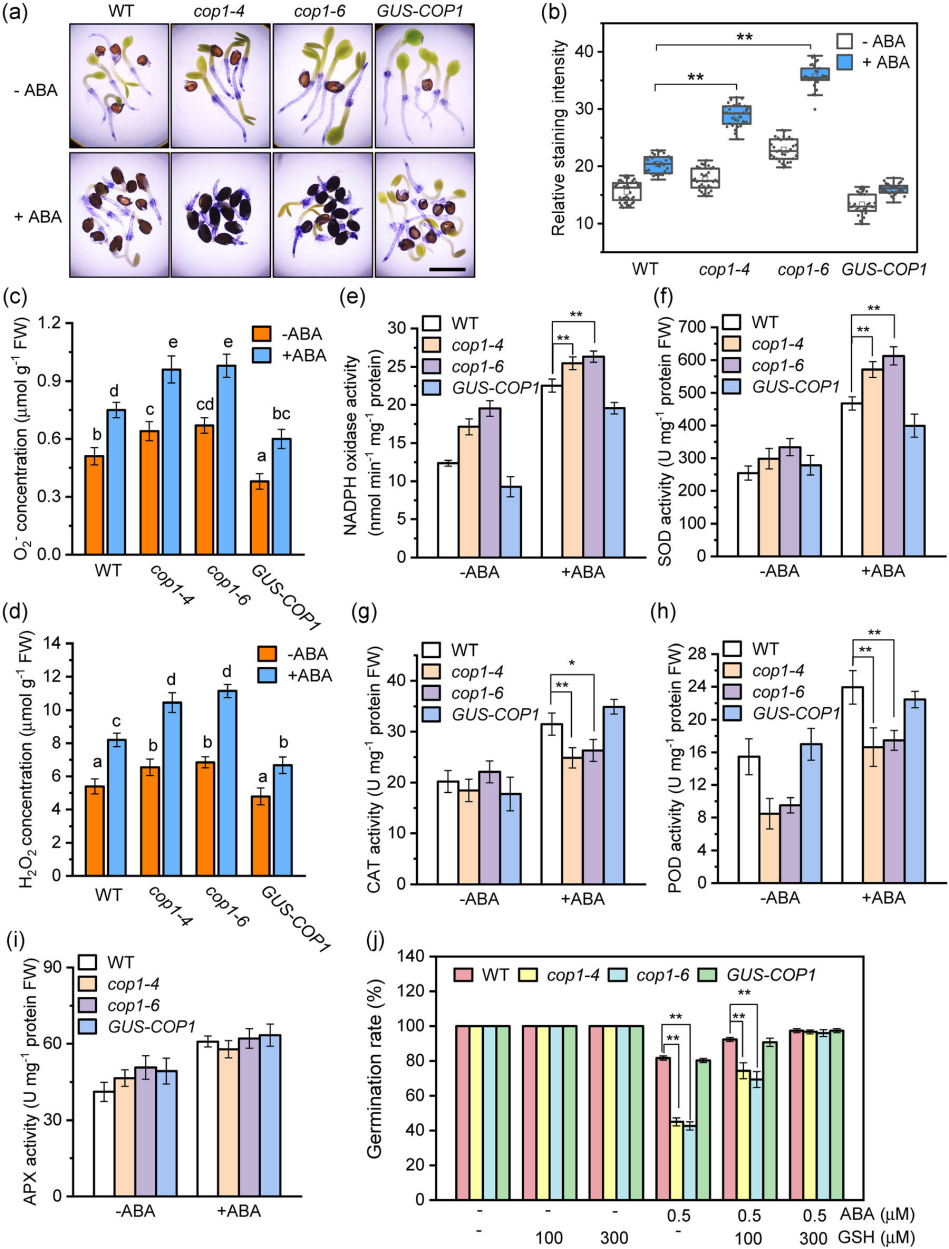
Loss of constitutive photomorphogenic 1 (COP1) enhances reactive oxygen species (ROS) accumulation in germinating seeds under abscisic acid (ABA) treatment. (a) Germinated seeds of *cop1* mutants showed deeper nitroblue tetrazolium (NBT) staining under ABA treatment than wild type (WT) seeds. WT, *cop1‐4*, *cop1‐6* and *GUS‐COP1* seeds were vernalized and germinated for 48 h on MS medium containing 0 or 0.5 μM ABA, before staining with NBT (scale = 0.5 mm).  (b) Relative staining intensity was measured on the seeds shown in (a). Statistical analyses were performed using a one‐way ANOVA. Data are means ± SD (*n* > 30 seeds per genotype), **p < 0.01. (c) and (d) O_2_^−^ and H_2_O_2_ concentration was measured in germinating seeds under the same conditions described in (a). Data are means ± SD of three replicates. Different letters indicate statistical differences at *p* < 0.05 (one‐way ANOVA analysis). (e–i) Enzyme activity values in germinating seeds under the same conditions described in (a). (e) NADPH‐oxidase, (f) superoxide dismutase (SOD), (g) catalase (CAT), (h) peroxidase (POD), and (i) ascorbate peroxidase (APX). Data are means ± SD of three replicates in one experiment. Three independent replicates were produced with similar results. Data are means ± SD, *n* = 3. *p < 0.05; **p < 0.01; student's *t*‐test. (j) Effect of the ROS scavenger GSH on ABA sensitivity in germinating seeds of WT, *cop1‐4*, *cop1‐6* and *GUS‐COP1*. Seeds were vernalized and germinated for 48 h on MS medium with different ABA and GSH concentrations. Data are means ± SD, *n* = 3. *p < 0.05; **p < 0.01; student's *t*‐test [Color figure can be viewed at wileyonlinelibrary.com]

External stimuli such as H_2_S, high salt stress, etc. and the phytohormone ABA can enhance ROS levels in seeds, inhibiting germination (Penfield, 2017). NBT staining of germinating seeds revealed stronger staining in *cop1‐4* and *cop1‐6* mutant seeds compared WT and *GUS‐COP1* under ABA treatment (Figure 2a,b). This suggests that disruption of COP1 leads to increased accumulation of ROS in the ABA‐mediated inhibition of seed germination. To verify the staining results, we quantified O_2_^−^ and H_2_O_2_ levels in germinating seeds of all genotypes in the presence or absence of ABA. Consistent with the NBT staining observations, the concentration of O_2_^−^ and H_2_O_2_ in seeds of *cop1* mutants were significantly higher than those of WT and *GUS‐COP1* under ABA treatment (Figure 2c,d). The activities of the ROS production‐related enzymes NADPH‐oxidase and superoxide dismutase (SOD) were significantly higher in *cop1* mutants under ABA treatment compared to WT (Figure 2e,f). In contrast, in the presence of ABA, the activities of the ROS scavenging related enzymes catalase (CAT) and peroxidase (POD) were significantly lower in *cop1‐4* and *cop1‐6* seeds than that in WT, while no significant differences were found in the activity values for ascorbate peroxidase (APX) (Figure 2g–i). These results suggest that COP1 reduces ABA‐induced ROS accumulation during seed germination.

To obtain additional evidence that the link between COP1 and ABA during seed germination is related to ROS, we measured the effect of adding the ROS scavenger glutathione (GSH) to germination medium containing 0.5 μM ABA. GSH is an important antioxidant in plants that can effectively reduce ROS accumulation in vivo. As observed earlier (Figure 1), germination rates on 0.5 μM ABA were significantly lower in *cop1* mutants compared to WT and *GUS‐COP1* lines (Figure 2j, Figure S2). Addition of 100 μM GSH to the ABA‐containing media partially decreased the hypersensitivity shown by *cop1* mutants while addition of 300 μM GSH to the media recovered germination rates in *cop1* mutants to WT levels (Figure 2j, Figure S2). These results suggest that GSH can alleviate the increased inhibitory effect of ABA on germination observed in *cop1* mutants.

### 3.3 ABA induces cytoplasmic accumulation of COP1 and increased ROS production

**Figure 3:**
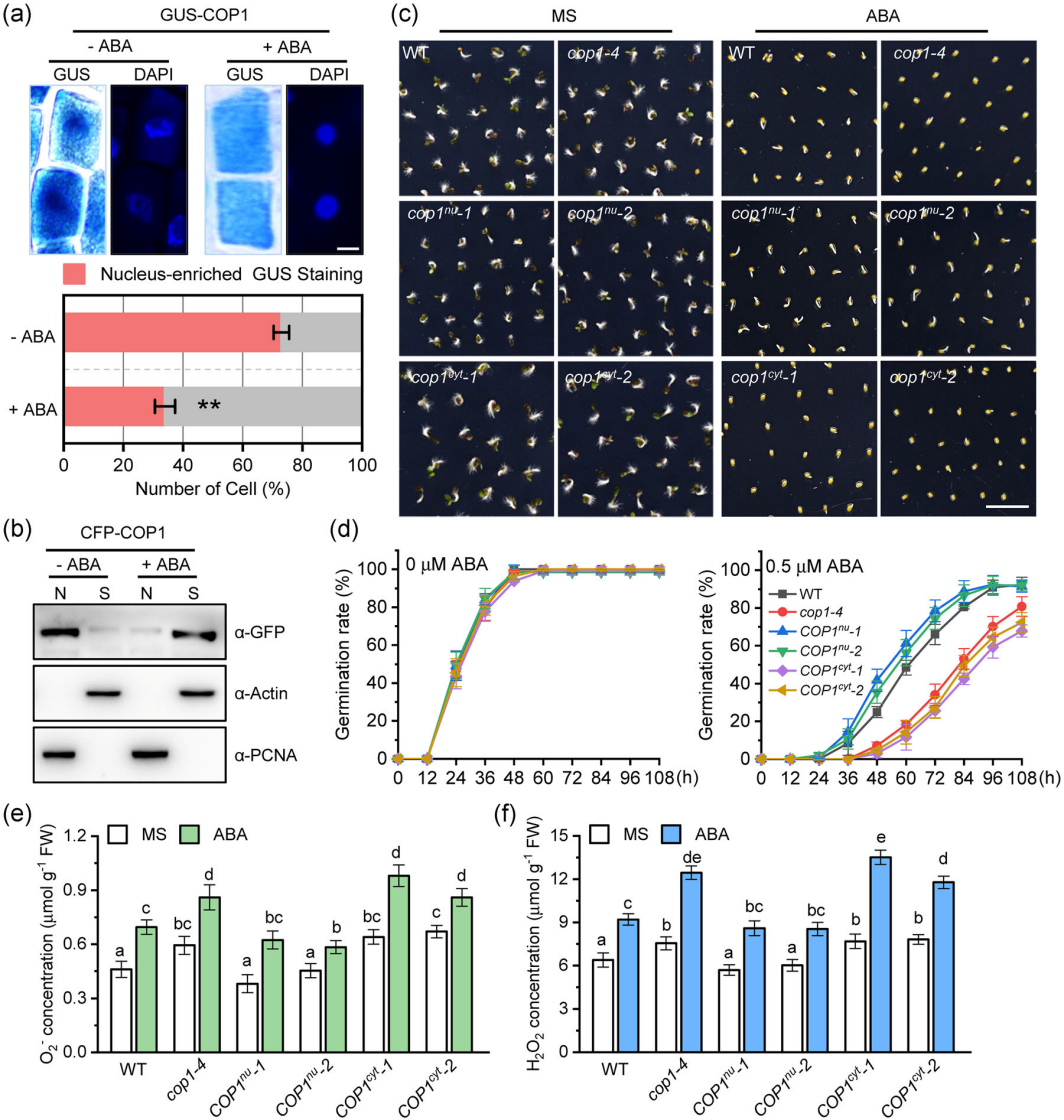
Cytoplasmic localization of constitutive photomorphogenic 1 (COP1) promotes abscisic acid (ABA)‐mediated inhibition of seed germination and ABA‐induced reactive oxygen species (ROS) accumulation. (a) *GUS‐COP1* seeds were sown on MS or MS supplemented with 0.5 μM ABA, vernalized, and germinated for 48 h. GUS staining was performed and the number of cells showing nuclear enriched staining was counted. Data are means ± SD, *n* = about 200 cells for three independent experiments. *p < 0.05; **p < 0.01; student's *t*‐test (scale = 1 μm). (b) Transgenic *CFP‐COP1* seeds were treated as described in (a), collected and proteins extracted. The relative abundance of CFP‐COP1 in cytoplasmic and nuclear fractions was detected using anti‐GFP antibodies. ACTIN was used as an internal reference for the presence of cytoplasmic proteins and proliferating cell nuclear antigen (PCNA) was used as an internal reference for the presence of nuclear proteins. N, nuclear fraction; S, soluble fraction containing cytoplasmic proteins. (c) Wild type (WT), *cop1‐4* mutants, COP1 nuclear‐localized mutants (*COP1* ^*nu*^ *−1*, *COP1* ^*nu*^ *−2*), and COP1 cytoplasm‐localized mutants (*COP1* ^*cyt*^ *−1*, *COP1* ^*cyt*^ *−2*) seeds were germinated on MS medium in the absence or presence of 0.5 μM ABA for 60 h (scale = 2 mm). (d) Germination rates for the seeds shown in (c). The time at which seeds were placed in the culture room was designated as 0 h, and the germination rates were measured every 12 h until 108 h. The values are means ± SD, (*n* > 50 seeds per genotype) of three replicates. (e) and (f) O_2_^−^ and H_2_O_2_ concentration was measured for the material shown in (c). Data are means ± SD of three replicates. Different letters indicate statistical differences at *p* < 0.05 (one‐way ANOVA analysis) [Color figure can be viewed at wileyonlinelibrary.com]

The nucleocytoplasmic partitioning of COP1 plays an important role in its function (Podolec & Ulm, 2018). Accumulation of COP1 in the nucleus results in the degradation of HY5, HYH and BBX21, inhibiting photomorphogenesis, while accumulation of COP1 in the cytoplasm enhances the stability of these transcription factors, promoting photomorphogenesis. To study the molecular mechanism by which COP1 affect ROS accumulation during ABA‐mediated inhibition of seed germination, we examined the effect of ABA on the nucleocytoplasmic partitioning of COP1 in germinating seeds. For this purpose, we used the *GUS‐COP1* line that express a GUS‐COP1 fusion protein. Staining of *GUS‐COP1* germinating seeds in the absence of ABA showed that GUS‐COP1 was significantly enriched in the nucleus with a large majority of cells (71%) showing enhanced nuclear staining compared to cytosol (Figure 3a). However, when seeds were germinated in the presence of 0.5 μM ABA, GUS staining was mostly distributed over the entire cell, and the number of cells with enriched nuclear staining was significantly lower (32.6%) (Figure 3a). For better showing the nucleocytoplasmic partitioning of COP1 to ABA treatment, we also used *CFP‐COP1* seeds. In agreement with the GUS staining results in *GUS‐COP1* seeds, CFP fluorescence observations in transgenic *CFP‐COP1* seeds showed that COP1 was predominantly located in the nucleus under normal condition for the majority of cells (83.1%) (Figure S3a,b). Treatment of *CFP‐COP1* seeds with ABA resulted in CFP fluorescence observed predominantly in the cytoplasm, with only a minority of cells (10.6%) showing COP1 enrichment in the nucleus (Figure S3a,b). These results suggest that ABA promotes the localization of COP1 to the cytoplasm during seed germination.

To complement our initial observations, we measured the relative amounts of CFP‐COP1 in nuclear and cytoplasmic protein fractions in transgenic *CFP‐COP1* seeds. Our results show that, in the absence of ABA, the majority of CFP‐COP1 is detected in the nuclear fraction of germinating seeds, while seeds germinated in the presence of 0.5 μM ABA contain most of the CFP‐COP1 protein in the cytoplasm (Figure 3b). These results suggest that ABA promotes COP1 accumulation in the cytoplasm during seed germination.

To explore whether the nucleocytoplasmic partitioning of COP1 mediates the ABA‐mediated inhibition of seed germination, we constructed COP1 nuclear‐localized mutants *COP1* ^*nu*^ and COP1 cytoplasm‐localized mutants *COP1* ^*cyt*^, and examined their germination rates under ABA treatment. COP1^nu^ contains a defective cytoplasmic localization signal (CLS), causing COP1 to localize in the nucleus (Figure S4) (Stacey et al., 1999, 2000). COP1^cyt^ contains a mutated nuclear localization signal (NLS), causing COP1 to localize in the cytoplasm (Figure S4) (Stacey et al., 1999, 2000). We expressed COP1^nu^ and COP1^cyt^ in the *cop1‐4* background obtaining two independent COP1 nuclear‐localized mutant lines *COP1* ^*nu*^ *−1*, *COP1* ^*nu*^ *−2* and two COP1 cytoplasm‐localized mutant lines *COP1* ^*cyt*^ *−1*, *COP1* ^*cyt*^ *−2* (Figure S5). All genotypes showed similar germination rates on MS medium (Figure 3c,d). Addition of 0.5 μM ABA to the germination medium had a stronger inhibitory effect on *cop1‐4*, *COP1* ^*cyt*^ *−1* and *COP1* ^*cyt*^ *−2* lines compared to WT, *COP1* ^*nu*^ *−1* and *COP1* ^*nu*^ *−2* lines, while the germination rates of two *COP1* ^*nu*^ lines were similar to those in WT with only slightly faster in some measurement time (Figure 3c,d), suggesting that the nucleocytoplasmic partitioning of COP1 affects the ABA‐mediated inhibition of seed germination, and the cytoplasmic localization of COP1 to ABA treatment enhances the sensitivity of seed germination.

To study the possible relationship between COP1 nucleocytoplasmic partitioning and ABA‐induced ROS during seed germination we measured O_2_^−^ and H_2_O_2_ levels in all genotypes in the absence and presence of ABA. Our results show that nuclear‐localized COP1 lines had similar O_2_^−^ and H_2_O_2_ levels than WT seeds (and lower than *cop1‐4* seeds), while O_2_^−^ and H_2_O_2_ levels in cytoplasmic COP1 lines were similar to those in *cop1‐4* seeds (and higher than WT) with or without ABA treatment (Figure 3e,f). In addition, the *RBOHD*, *RBOHF* and *SOD2* expression levels in *COP1* ^*cyt*^ *−1* and *COP1* ^*cyt*^ *−2* seeds were also higher than those in *COP1* ^*nu*^ *−1* and *COP1* ^*nu*^ *−2* seeds (Figure S6). While among these three genes, only *SOD2* expression levels in *COP1* ^*nu*^ lines are similar to those in WT (Figure S6). To further clarify the causal relationship between ABA‐induced COP1 cytoplasmic localization and ABA‐induced ROS, we tested the effect of the active oxygen scavenger GSH on the localization of COP1. GSH significantly reduced the accumulation of O_2_^−^ and H_2_O_2_ induced by ABA in *CFP‐COP1* seeds (Figure S3c,d), but GSH did not significantly change the cytoplasmic localization of CFP‐COP1 in *CFP‐COP1* seeds under ABA treatment (Figure S3a,b). Therefore, these results indicate that ABA‐induced ROS production is not the cause for the cytoplasmic localization of COP1 during ABA treatment. Overall, our results indicate that ABA induces the accumulation of COP1 in the cytoplasm during seed germination, which results in higher ROS levels in the seed.

### 3.4 The HY5‐ABI5 pathway regulates the effect of COP1 in the ABA‐mediated inhibition of seed germination and ROS accumulation

**Figure 4:**
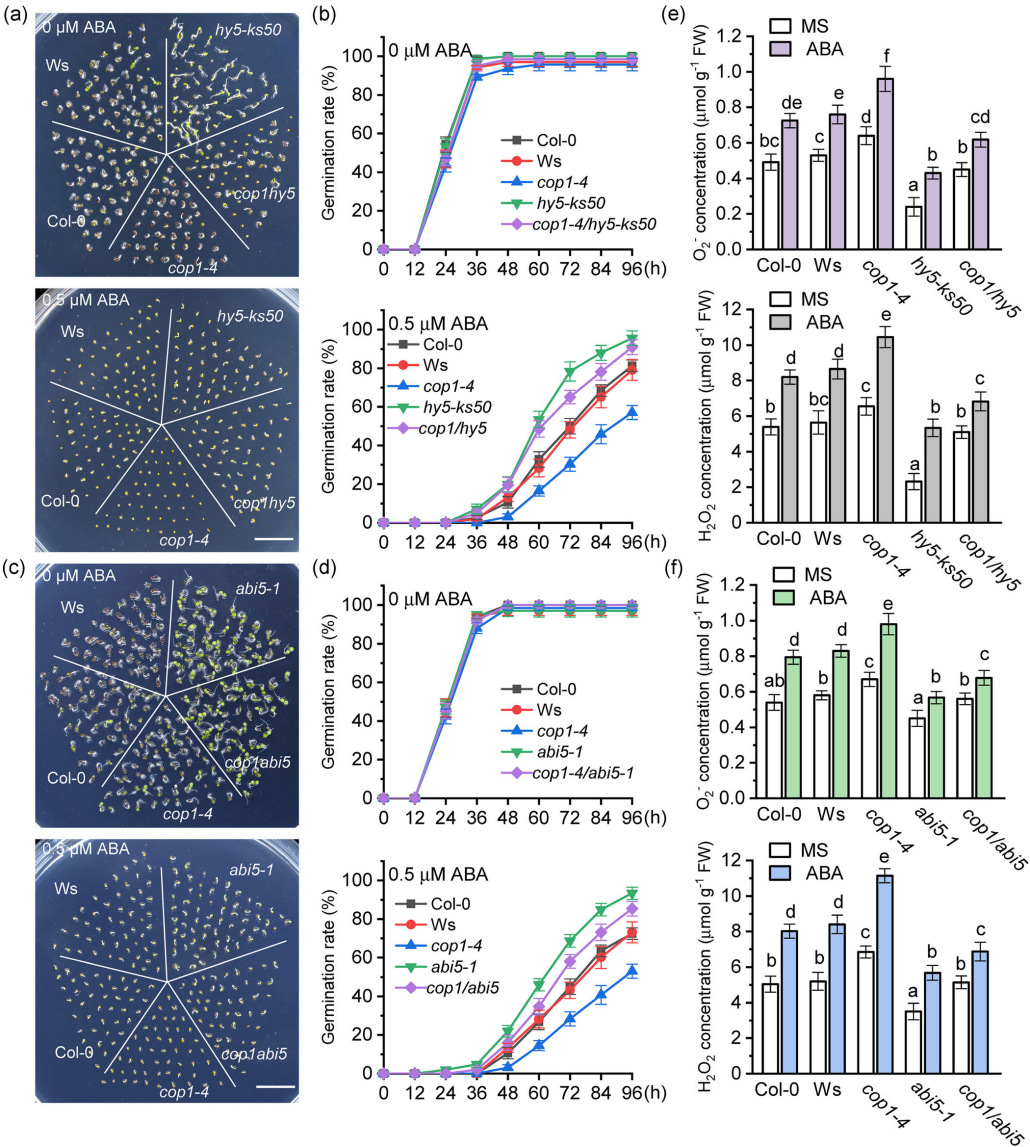
Mutations in *HY5 *and *ABI5 *reduces abscisic acid (ABA) sensitivity and ABA‐induced reactive oxygen species (ROS) accumulation in*cop1‐4*seeds. (a) Col‐0, *cop1‐4*, *cop1‐4/hy5‐ks50*, *hy5‐ks50* and Ws seeds germinated on MS plates containing 0 or 0.5 μM ABA for 48 h (scale = 1 cm). (b) Germination rates of the genotypes shown in (a). The time at which seeds were placed in the culture room was designated as 0 h, and the germination rates were measured every 12 h until 96 h. Data are means ± SD, (*n* > 50 seeds per genotype) of three replicates. (c) Col‐0, *cop1‐4*, *cop1‐4/abi5‐1*, *abi5‐1* and Ws seeds germinated on MS or MS supplemented with 0.5 μM ABA for 48 h (scale = 1 cm). (d) Germination rates of the genotypes shown in (c). The time at which seeds were placed in the culture room was designated as 0 h, and the germination rates were measured every 12 h until 96 h. Data are means ± SD, (*n* > 50 seeds per genotype) of three replicates. (e) O_2_^−^ and H_2_O_2_ concentration was measured for the material shown in (a). Data are means ± SD of three replicates. Different letters indicate statistical differences at *p* < 0.05 (one‐way ANOVA analysis). (f) O_2_^−^ and H_2_O_2_ concentration was measured for the material shown in (c). Data are means ± SD of three replicates. Different letters indicate statistical differences at *p* < 0.05 (one‐way ANOVA analysis) [Color figure can be viewed at wileyonlinelibrary.com]

Previous studies have shown that HY5 regulates ABA‐mediated inhibition of seed germination by enhancing ABI5 expression through binding to the ABI5 promoter or direct interaction with ABI5 (Chen et al., 2008; Wang et al., 2021). HY5 and ABI5 have also been linked to ROS production (Bi et al., 2017; Chen et al., 2013; Wang et al., 2018), raising the posibility that the HY5‐ABI5 pathway could regulate ROS accumulation during ABA inhibition of seed germination. To explore whether the role of COP1 in the ABA‐induced ROS accumulation in seeds is mediated by the HY5‐ABI5 pathway, we obtained and analysed *cop1‐4/hy5‐ks50* and *cop1‐4/abi5‐1* double mutants (Figure 4, Figures S7 and S8). *Arabidopsis* seeds from Col‐0, Ws, *cop1‐4*, *hy5‐ks50* and *cop1‐4/hy5‐ks50* had similar germination rates on MS medium, but differences were apparent in medium containing 0.5 μM ABA (Figure 4a,b). Single *cop1‐4* mutant seeds showed the strongest ABA inhibition while *hy5‐ks50* mutants showed the lowest ABA inhibition. The two WT ecotypes, Col‐0 and Ws, were identical to each other and showed an intermediate level of ABA sensitivity. Importantly, the germination rate of *cop1‐4/hy5‐ks50* double mutants was higher than that of the *cop1‐4* single mutant indicating that mutations in *HY5* suppress the hypersensitivity of *cop1‐4* to ABA‐mediated inhibition of seed germination (Figure 4a,b). Analysis of Col‐0, Ws, *cop1‐4*, *abi5‐1* and *cop1‐4/abi5‐1* showed very similar results, with mutations in *ABI5* (*cop1‐4/abi5‐1*) reversing the ABA hypersensitivity shown by *cop1‐4* mutants (Figure 4c,d). Our genetic evidence indicates that COP1 regulates ABA inhibition of seed germination through the HY5‐ABI5 pathway.

To study whether the COP1‐HY5‐ABI5 pathway mediates ABA‐promoted ROS accumulation during seed germination, we measured O_2_^−^ and H_2_O_2_ levels in all genotypes under normal and ABA treatment conditions. The O_2_^−^ and H_2_O_2_ levels in seeds of *hy5‐ks50* mutants were significantly lower than all other genotypes under normal and ABA conditions, while *cop1‐4* mutants showed higher O_2_^−^ and H_2_O_2_ levels than those of other genotypes (Figure 4e). Notably, O_2_^−^ and H_2_O_2_ levels in *cop1‐4/hy5‐ks50* double mutant seeds were significantly lower than those in *cop1‐4* seeds (Figure 4e), suggesting that the absence of HY5 reduced ROS accumulation in *cop1‐4* seeds. These observations support the germination results (Figure 4a,b). Similarly, the O_2_^−^ and H_2_O_2_ levels in *cop1‐4/abi5‐1* seeds were significantly lower than those of *cop1‐4* seeds under ABA treatment (Figure 4f), suggesting that the *ABI5* mutation also reduces ROS accumulation in *cop1‐4* seeds. In conclusion, these results indicate COP1 can regulate ABA‐induced ROS accumulation through the HY5‐ABI5 pathway during ABA‐mediated inhibition of seed germination.

### 3.5 ABA‐induced cytoplasmic localization of COP1 enhances activation of the nuclear HY5‐ABI5 pathway

**Figure 5:**
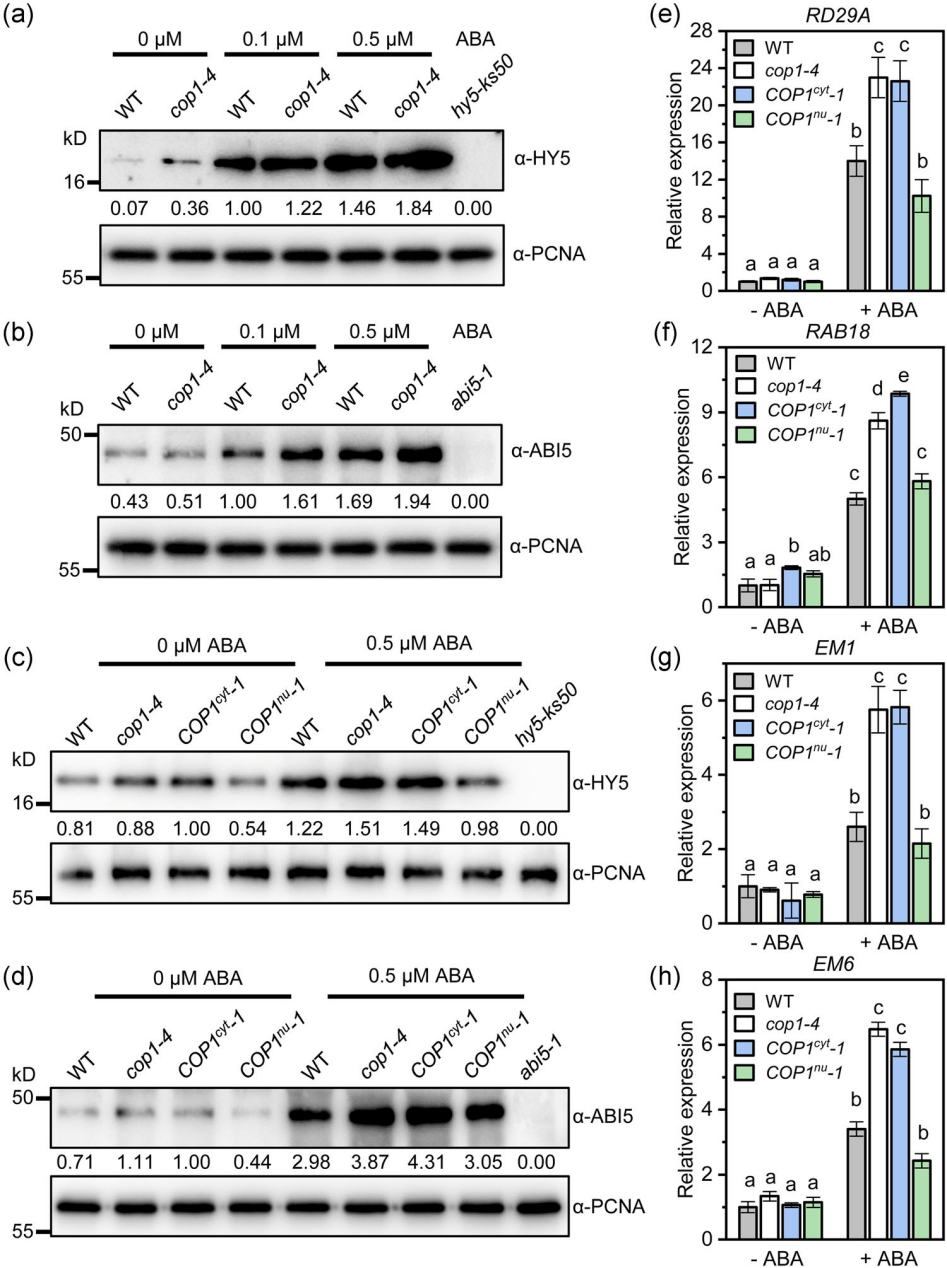
Abscisic acid (ABA)‐induced accumulation of constitutive photomorphogenic 1 (COP1) in the cytoplasm enhances the activity of the HY5‐ABI5 pathway during ABA‐mediated inhibition of seed germination. (a) Western blot analysis of HY5 protein levels in seeds of wild type (WT) and *cop1‐4* germinated on 0, 0.1 or 0.5 μM ABA medium for 36 h using HY5 antibodies (AS12 1867; Agriser Corp.). *hy5‐ks50* seeds were used as negative control. Proliferating cell nuclear antigen (PCNA) protein was used as an internal reference. The numbers represent the relative intensity of the HY5 protein band normalized versus the respective PCNA band. Three independent experiments were performed with similar results. (b) Western blot analysis of ABI5 protein levels in seeds of WT and *cop1‐4* germinated on 0, 0.1 or 0.5 μM ABA medium for 36 h using ABI5 antibodies (AS12 1863; Agrisera Corp.). *abi5‐1* seeds were used as negative control. PCNA protein was used as an internal reference. The numbers represent the relative intensity of the ABI5 protein band normalized versus the respective PCNA band. Three independent experiments were performed with similar results. (c) Western blot analysis of HY5 protein levels in seeds of WT, *cop1‐4*, COP1 cytoplasm‐localized mutant *COP1* ^*cyt*^ *−1* and COP1 nuclear‐localized mutant *COP1* ^*nu*^ *−1* germinated on 0 and 0.5 μM ABA medium for 36 h. *hy5‐ks50* seeds were used as negative control. PCNA protein was used as an internal reference. The numbers represent the relative intensity of the HY5 protein band normalized versus the respective PCNA band. Three independent experiments were performed with similar results. (d) Western blot analysis of ABI5 protein levels in seeds of WT, *cop1‐4*, *COP1* ^*cyt*^ *−1* and *COP1* ^*nu*^ *−1* seeds under the same treatment conditions as in (c). *abi5‐1* seeds were used as negative control. PCNA protein was used as an internal reference. The numbers represent the relative intensity of the ABI5 protein band normalized versus the respective PCNA band. Three independent experiments were performed with similar results. (e–h) Expression levels of *RD29A* (e), *RAB18* (f), *EM1* (g), *EM6* (h) genes in WT, *cop1‐4*, *COP1* ^*cyt*^ *−1* and *COP1* ^*nu*^ *−1* seeds were detected by qRT‐PCR. The values are means ± SD of three biological replicates. Different letters indicate statistical differences at *p* < 0.05 (one‐way ANOVA analysis) [Color figure can be viewed at wileyonlinelibrary.com]

Our results so far have shown that ABA induces COP1 accumulation in cytoplasm during seed germination while genetic evidence suggests that the role of COP1 in the ABA‐mediated inhibition of germination is exerted through the HY5‐ABI5 pathway. We therefore hypothesized that, during seed germination, the ABA‐induced cytoplasmic accumulation of COP1 could increase HY5 and/or ABI5 levels, thus activating the HY5‐ABI5 pathway. To test this hypothesis, we first examined the effects of the absence of COP1 on HY5 and ABI5 protein levels in response to ABA during seed germination. Western blot analysis of germinating WT and *cop1‐4* seeds in the absence or presence of ABA was performed and the HY5 protein levels visualized. As expected, the presence of ABA increased HY5 levels in WT seeds (Figure 5a). Notably, the ABA‐induced HY5 levels in *cop1‐4* seeds were always higher than those observed in WT seeds. When the same experiment was performed using antibodies against ABI5 we observed that ABA increased ABI5 levels in WT seeds, but the increase was more pronounced in *cop1‐4* seeds suggesting that loss of COP1 enhances the ABA‐induced increase in ABI5 levels (Figure 5b). qPCR showed that ABA induction of the *ABI5* expression levels was far more pronounced in *cop1‐4* and *cop1‐6* mutants than in WT seeds (Figure S9a).

To investigate the effect of COP1 localization on the HY5 and ABI5 protein levels in response to ABA treatment, we analysed the cytoplasm‐ and nuclear‐localized mutants, *COP1* ^*cyt*^ *−1* and *COP1* ^*nu*^ *−1* respectively. Western blot analysis of germinating seeds in the presence or absence of ABA revealed that the enhanced HY5 and ABI5 levels observed in *cop1‐4* mutants versus WT seeds was also observed in cytoplasmic localized *COP1* ^*cyt*^ *−1* lines but was not present in nuclear‐localized *COP1* ^*nu*^ *−1* lines (Figure 5c,d). In addition, *ABI5* transcript levels in response to ABA were higher in *cop1‐4* and *COP1* ^*cyt*^ *−1* seeds than in WT while *COP1* ^*nu*^ *−1* seeds showed WT levels (Figure S9b). These results suggest that accumulation of COP1 in the cytoplasm enhances the ABA‐induced increase of HY5 and ABI5 protein levels.

To further examine the effect of COP1 localization on the ABA activation of the HY5‐ABI5 pathway, we examined the expression levels of several ABI5 target genes (*RD29A*, *RAB18*, *EM1* and *EM6*) in WT, *cop1‐4*, *COP1* ^*cyt*^ *−1*, and *COP1* ^*nu*^ *−1* seeds under ABA treatment. The results showed that, for all analysed genes, ABA consistently induced higher expression levels in *cop1‐4* and *COP1* ^*cyt*^ *−1* seeds compared to WT seeds, while *COP1* ^*nu*^ *−1* seeds showed similar levels of induction than WT (Figure 5e–h). This suggests that the ABA‐induced accumulation of COP1 in the cytoplasm enhances the activity of the HY5‐ABI5 pathway during ABA‐mediated inhibition of seed germination.

### 3.6 The higher ABA‐induced ROS levels in cytoplasmic COP1 lines is dependent on the HY5‐ABI5 pathway

**Figure 6:**
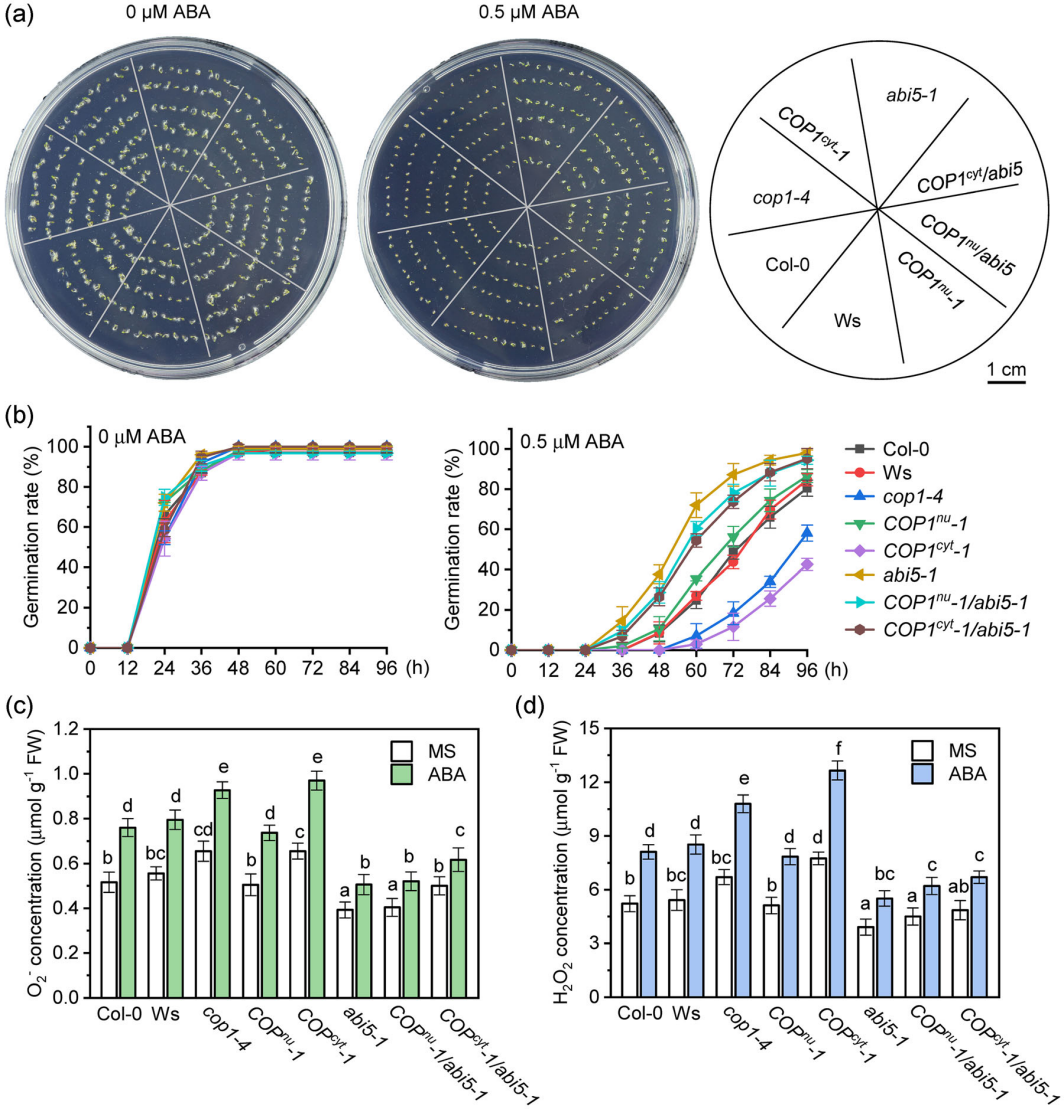
ABI5 is involved in the regulation of reactive oxygen species (ROS) accumulation promoted by abscisic acid (ABA)‐induced constitutive photomorphogenic 1 (COP1) cytoplasmic localization. (a) Col‐0, Ws, *cop1‐4*, *COP1* ^*nu*^ *−1*, *COP1* ^*cyt*^ *−1*, *abi5‐1*, *COP1* ^*nu*^ *−1/abi5‐1*, *COP1* ^*cyt*^ *−1/abi5‐1* seeds germinated for 48 h on MS plates containing 0 μM ABA or 0.5 μM ABA. (b) Germination rates of the genotypes shown in (a). The time at which seeds were placed in the culture room was designated as 0 h, and the germination rates were measured every 12 h until 96 h. Data are means ± SD, (*n* > 50 seeds per genotype) of three replicates. (c) and (d) O_2_^−^ concentration (c) and H_2_O_2_ concentration (d) of the genotypes shown in (a). The values are means ± SD of three replicates. Different letters indicate statistical differences at *p* < 0.05 (one‐way ANOVA analysis) [Color figure can be viewed at wileyonlinelibrary.com]

To study whether the increased ABA‐induced ROS levels observed in cytoplasmic COP1 localized lines is dependent on the HY5‐ABI5 pathway, we produced and analysed double *COP1* ^*nu*^ *−1/abi5‐1* and *COP1* ^*cyt*^ *−1/abi5‐1* mutants (Figure 6, Figure S10). In the absence of ABA, Col‐0, Ws, *cop1‐4*, *COP1* ^*nu*^ *−1*, *COP1* ^*cyt*^ *−1*, *abi5‐1*, *COP1* ^*nu*^ *−1/abi5‐1*, *COP1* ^*cyt*^ *−1/abi5‐1* seeds showed similar germination rates (Figure 6a,b). In agreement with our previous results, *cop1‐*4 and *COP1* ^*cyt*^ *−1* seeds show enhanced ABA sensitivity compared to WT (Col‐0 and Ws) (Figure 6a,b) while *COP1* ^*nu*^ *−1* seeds show slightly lower sensitivity than WT. Importantly, the enhanced ABA sensitivity shown by *COP1* ^*cyt*^ *−1* seeds disappeared in the double *COP1* ^*cyt*^ *−1/abi5‐1* mutants (Figure 6a,b) indicating that the ABA hypersensitivity of *COP1* ^*cyt*^ *−1* seeds is mediated by ABI5.

Analysis of O_2_^−^ and H_2_O_2_ levels in all genotypes showed lower ROS levels in *abi5‐1*, *COP1* ^*nu*^ *−1/abi5‐1* and *COP1* ^*cyt*^ *−1/abi5‐1* seeds (Figure 6c,d) suggesting that the lower accumulation of ABA‐induced ROS in these genotypes is the cause for their increased insensitivity to ABA relative to other genotypes. Notedly, the ABA‐induced O_2_^−^ and H_2_O_2_ levels in seeds of the COP1 cytoplasm‐localized mutant *COP1* ^*cyt*^ *−1* were higher than those of other genotypes but those levels were significantly reduced in the *COP1* ^*cyt*^ *−1/abi5‐1* double mutant (Figure 6c,d), suggesting that ABI5 is involved in the enhanced ROS accumulation in response to ABA observed in the cytosolic localized COP1 seeds.

## 4 DISCUSSION

In the life cycle of flowering plants, seed germination is the first and crucial step determining the beginning of plant life. The transition from seed dormancy to germination is tightly regulated by a comprehensive set of internal signals and environmental stimuli. The phytohormone ABA, as an endogenous signal, activates a series of downstream physiological and biochemical reactions to inhibit seed germination (Bahin et al., 2011; Bailly, 2019; Oracz & Karpinski, 2016), while light acts as an environmental signal by balancing the ABA/GA ratio to regulate seed germination (Seo et al., 2009). In addition, the light signalling component COP1 can directly participate in ABA‐mediated developmental processes such as stomatal movement (Chen et al., 2021), but its role in ABA inhibition of seed germination is unclear.

This study provides several lines of evidence strongly supporting a pivotal role for COP1 in the ABA‐mediated inhibition of seed germination. Firstly, disruption of COP1 enhances sensitivity to ABA and raises ABA‐induced ROS levels in seeds (Figures 1 and 2). Second, COP1 substrates HY5 and ABI5, positively regulate ABA‐mediated inhibition of seed germination, and mutations in *HY5* and *ABI5* effectively suppress *cop1‐4* hypersensitivity to ABA during seed germination and the enhanced ABA‐induced ROS levels observed in *cop1‐4* seeds (Figure 4). Overall, the evidence suggests that COP1 is a negative modulator of the ABA‐mediated inhibition of seed germination via the HY5‐ABI5 pathway.

COP1, as an E3 ligase, marks for degradation a number of regulators involved in light and other signalling pathways, and its function is always affected by its nucleocytoplasmic partitioning (Podolec & Ulm, 2018; Ponnu & Hoecker, 2021). Enhanced nuclear localization of COP1 by elevated temperatures facilitates hypocotyl growth by increasing HY5 degradation (Park et al., 2017). In contrast, jasmonic acid reduces the amount of nuclear‐localized COP1 promoting the suppression of hypocotyl elongation (Zheng et al., 2017). Under some abiotic stress conditions, such as high salt and high temperature, COP1 is induced to accumulate in the cytoplasm suggesting that the cytoplasmic localization of COP1 promotes inhibition of seed germination (Chen et al., 2019; Yu et al., 2016). We observed that treatment of seeds with ABA enhances COP1 localization in the cytoplasm. The hypersensitivity to ABA shown by cytoplasmic *COP1* ^*cyt*^ lines suggests that the cytoplasmic localization of COP1 enhances the inhibitory effect of ABA on seed germination. COP1's E3 ligase function requires the participation of SPA proteins (Podolec & Ulm, 2018). Similar to *cop1* mutants, *spa1* mutants were also hypersensitive to ABA during seed germination (Gangappa et al., 2010), suggesting that the COP1/SPAs complex play a regulatory role in ABA‐mediated inhibition of seed germination. Therefore, the cytoplasmic accumulation of COP1, induced by ABA, may result in increased degradation of cytoplasmic proteins enhancing the ABA‐mediated inhibition of seed germination. Although SPAs and FIN219 have been identified as factors modulating the nucleocytoplasmic partitioning of COP1 (Balcerowicz et al., 2017; Swain et al., 2017), the mechanism that regulates this partitioning is still unclear.

ROS are key signalling intermediates involved in a wide range of plant responses and developmental processes (Bailly, 2019; Ros Barcelo & Gomez Ros, 2009). HY5 acts as the bridge between light and ROS signalling to regulate protochlorophyllide synthesis and cell death in the light during seeding establishment (Bellegarde et al., 2019; Chai et al., 2015; Chen et al., 2013) and can bind to the promoter of several ROS‐responsive genes modulating their expression (Chen et al., 2013). ABI5 plays an important role regulating ROS balance during germination, with *abi5* mutants exhibiting altered expression of genes involved in ROS metabolism and response, and impaired ROS signalling during ABA‐mediated inhibition of seed germination (Bi et al., 2017). Although high ROS levels had been previously reported in *cop1* mutants (Xu et al., 2016), the role of COP1 regulating ABA‐induced ROS accumulation during seed germination remained unclear. Our results show that *cop1* mutant seeds produce increased amounts of O_2_^−^ and H_2_O_2_ under ABA treatment (Figure 2c,d), while mutations in HY5 and ABI5 effectively reduced the levels of O_2_^−^ and H_2_O_2_ in *cop1* seeds, suggesting that HY5 and ABI5 mediate the inhibitory role of COP1 in the ABA‐induced ROS accumulation during seed germination (Figure 4e,f). However, ROS levels in seeds of *cop1*/*hy5* and *cop1*/*abi5* double mutants were still significantly higher than those of *hy5* and *abi5* single mutants suggesting that the effect of COP1 on ROS accumulation to ABA is not completely dependent on HY5 and ABI5 (Figure 4e,f).

The nucleocytoplasmic partitioning of COP1 had a significant effect on ABA‐induced ROS accumulation during seed germination. The seeds of cytoplasm localized *COP1* ^*cyt*^ lines accumulated higher ROS levels (Figure 3e,f), and increased HY5 and ABI5 protein levels than the nuclear‐localized *COP1* ^*nu*^ lines and WT under ABA treatment (Figure 5c,d), suggesting that localization of COP1 in the cytoplasm is conducive to the ABA‐induced increase in ROS, HY5 and ABI5 levels. While, the localization of COP1 in the nuclear accumulated similar ROS levels to those in WT to ABA treatment, and which are in accordance with their final similar germination (Figure 3d–f). ABA treatment induced higher expression levels of ABI5 target genes in *COP1* ^*cyt*^ seeds compared to WT and *COP1* ^*nu*^ (Figure 5e–h), and mutation of ABI5 was able to reduce the ROS levels in *COP1* ^*cyt*^ seeds (Figure 6c,d). These results indicate that ABI5 acts as a downstream regulator enhancing the production of ROS after the ABA‐induced cytoplasmic accumulation of COP1. Notably, the ROS scavenger GSH can effectively reduce ABA‐induced ROS accumulation in *CFP‐COP1* seeds (Figure S3c,d), but does not impair ABA‐induced COP1 cytoplasmic localization (Figure S3a,b), implying ABA‐induced cytoplasmic accumulation of COP1 is not caused by ABA‐induced ROS production during seed germination. Therefore, we hypothesize that ABA induces COP1 export from the nucleus to the cytosol, increasing HY5 and ABI5 levels in the nucleus and promoting the expression of ROS‐responsive genes.

While our work shows that COP1 negatively regulates ABA‐mediated inhibition of seed germination, previous reports identified COP1 as a positive factor regulating ABA inhibition after‐germination (Lee et al., 2021; Yadukrishnan et al., 2020). These results suggest that COP1 has different regulatory roles in these two ABA‐mediated processes. Under ABA treatment, *cop1* mutants exhibited hypersensitivity to ABA during seed germination, but hyposensitivity to ABA in seeding establishment. Our results show that disruption of COP1 enhances seed sensitivity to ABA, probably caused by increased accumulation ABI5 protein (Figure 5b), whereas the insensitivity of *cop1* mutants to ABA‐mediated inhibition of post‐germination was caused by reduced ABI5 protein levels (Yadukrishnan et al., 2020). Overall, it seems that COP1 involvement in these two ABA‐mediated processes is exerted by regulating ABI5 protein levels. However, the detailed regulatory mechanism remains to be elucidated.

**Figure 7:**
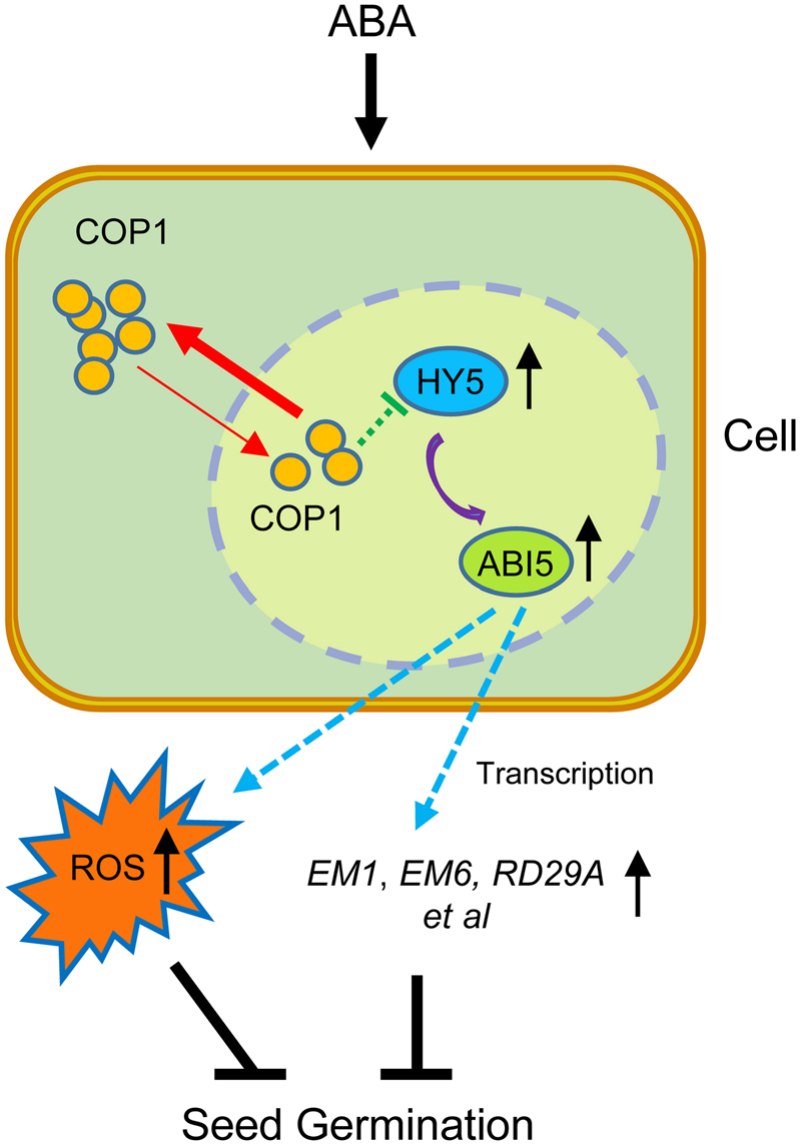
Model for the involvement of constitutive photomorphogenic 1 (COP1) in abscisic acid (ABA)‐mediated inhibition of seed germination in *Arabidopsis*. During *Arabidopsis* seed germination, ABA promotes COP1 export from the nucleus and accumulation in the cytoplasm. The reduction of COP1 levels in the nucleus enhances the HY5 stability increasing HY5 levels in the nucleus. Increase nuclear HY5 levels promote *ABI5* gene expression leading to the accumulation of ABI5. Accumulation of ABI5 enhances ABA‐induced reactive oxygen species (ROS) levels and promotes the expression of ABA‐related genes, thus inhibiting seed germination of *Arabidopsis thaliana*. The red arrow line represents the transfer direction of COP1. The green blocking dotted line represents the weakened ubiquitination‐mediated degradation of HY5 by reduced levels of nuclear COP1. The upward black arrows represent increased protein levels or gene expression. The purple curved arrow represents the accumulation of HY5‐induced ABI5. The dotted line on the blue arrow indicates the ABI5‐enhanced ROS accumulation and downstream gene expression [Color figure can be viewed at wileyonlinelibrary.com]

We propose a mechanistic model in which, during seed germination, ABA induces COP1 export to cytoplasm (Figure 7). The reduced nuclear levels of COP1 result in decreased degradation of HY5, increasing the levels of HY5 and, as a consequence, the levels of ABI5 in the nucleus. For exploring whether COP1 can directly degrade ABI5, the ubiquitination experiment of COP1 on ABI5 needs to be designed in the future research. Here, the enhanced ABI5 levels promote the transcription of multiple ABA‐responsive genes as well as the levels of ABA‐induced ROS, both of which have a detrimental effect on seed germination (Figure 7). The elucidation of this mechanism explains the role of COP1 in ABA‐mediated inhibition of seed germination, and places COP1 as an integrator of the crosstalk between light and ABA signalling.

## SUMMARY STATEMENT

COP1, the core repressor of light signals, functions in ABA‐mediated inhibition of *Arabidopsis* seed germination. The ABA‐induced COP1 cytoplasmic translocation increases both HY5 and ABI5 protein levels in the nucleus, leading to elevated expression of ABI5 target genes and enhanced ROS levels.

## CONFLICTS OF INTEREST

The authors declare no conflicts of interest.

## Supporting information

Supporting information.

Supporting information.

Supporting information.

Supporting information.

Supporting information.

Supporting information.

Supporting information.

Supporting information.

Supporting information.

Supporting information.

Supporting information.

## Data Availability

Data is available on request from the authors.
