# Supplementary figures and images for: Abscisic acid‐induced cytoplasmic translocation of constitutive photomorphogenic 1 enhances reactive oxygen species accumulation through the HY5‐ABI5 pathway to modulate seed germination
Source: Plant Cell Environ. 2022 Mar 10;45(5):1474–89. doi: 10.1111/pce.14298 (PMC9311139; doi:10.1111/pce.14298)

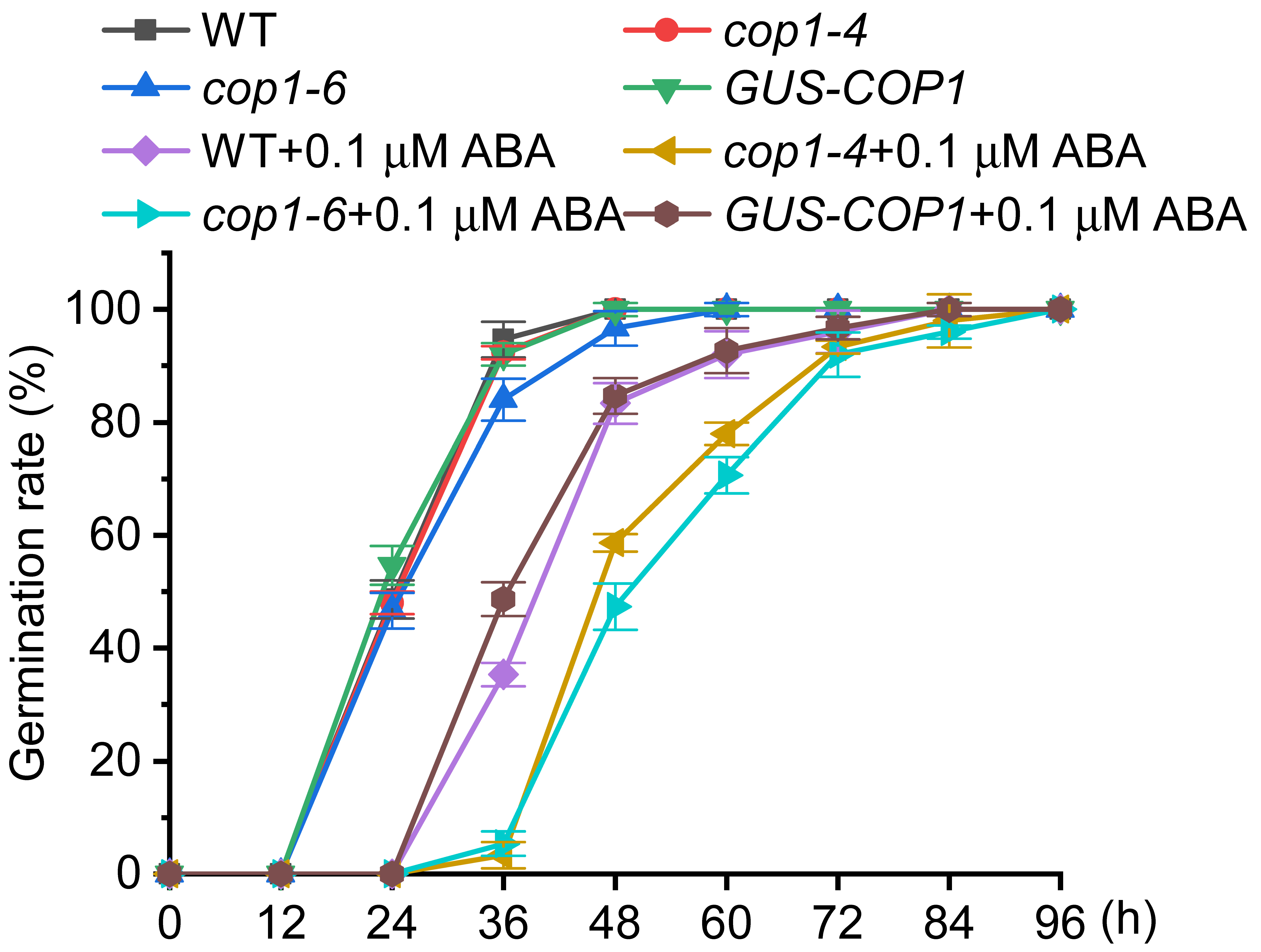

Supplement: Supplementary file 1 — Supporting information. [file PCE-45-1474-s005.jpg]

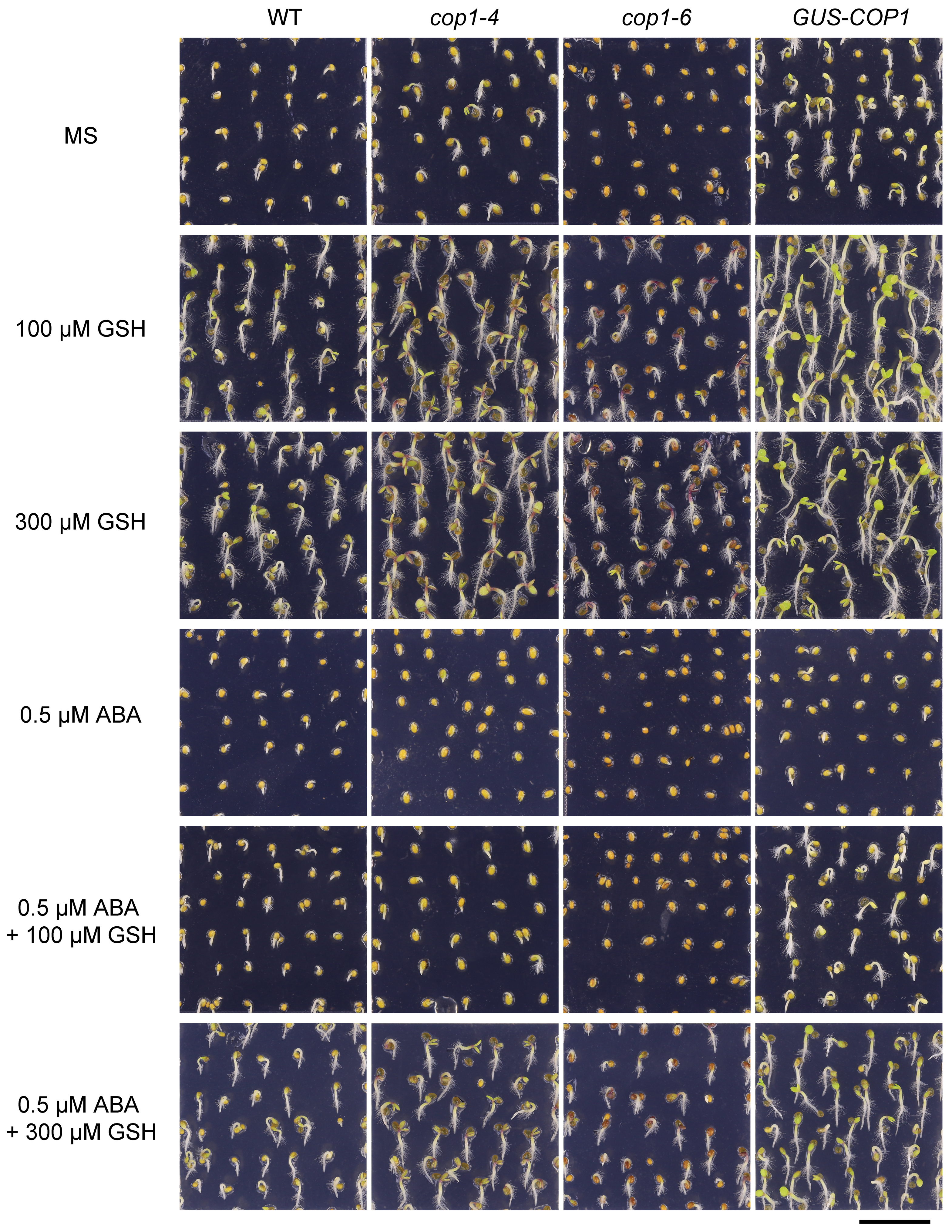

Supplement: Supplementary file 2 — Supporting information. [file PCE-45-1474-s008.jpg]

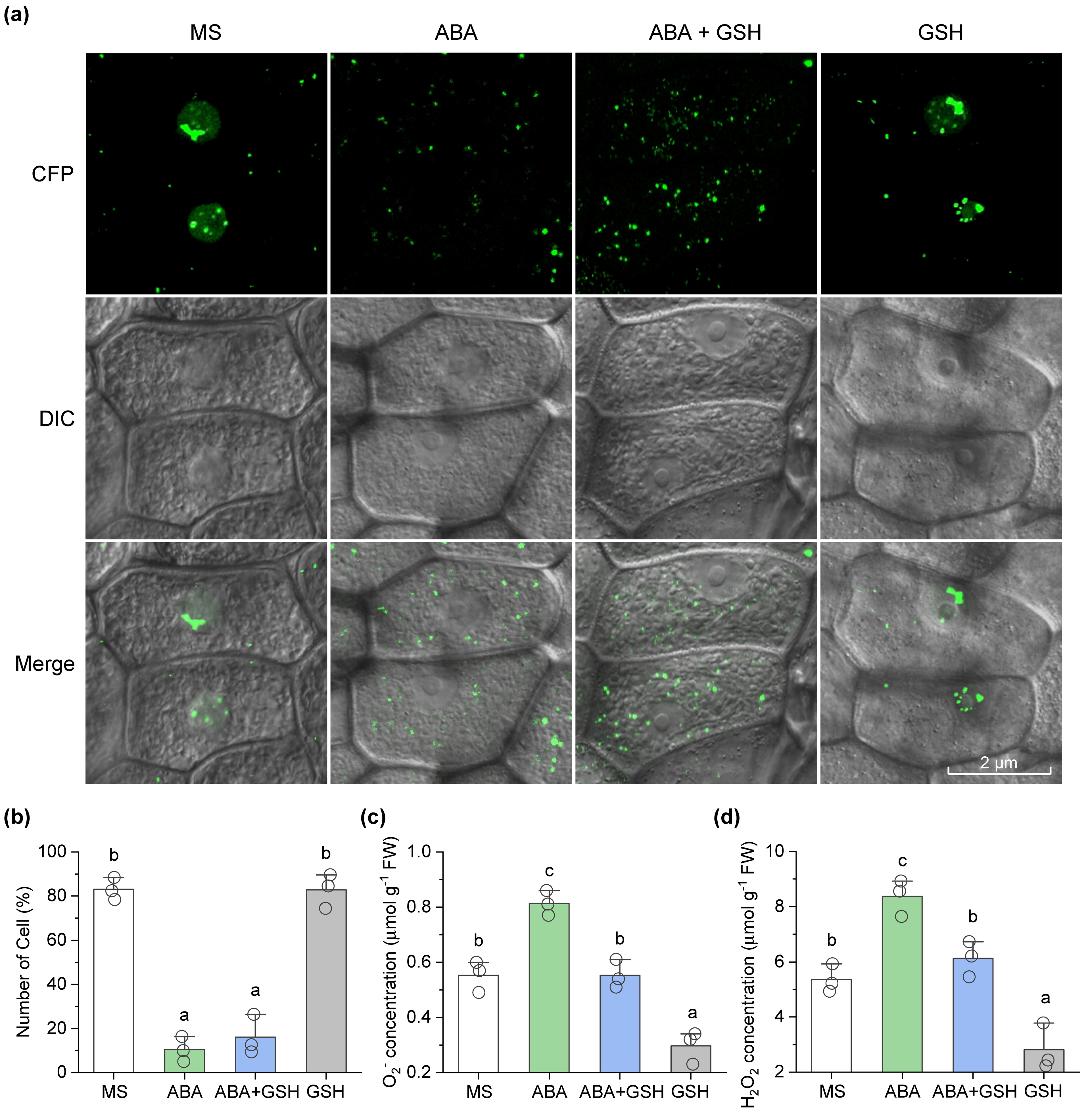

Supplement: Supplementary file 3 — Supporting information. [file PCE-45-1474-s011.jpg]

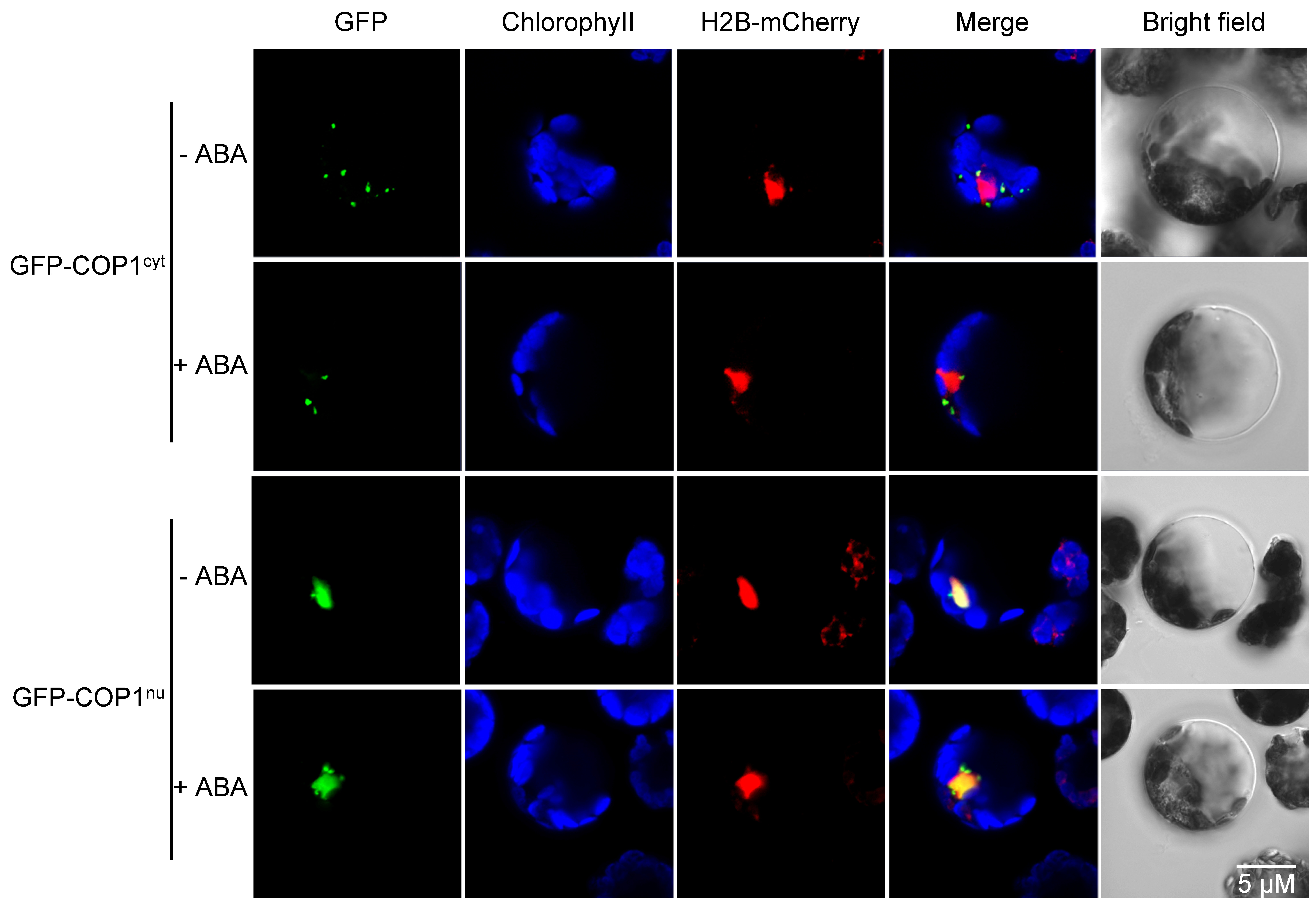

Supplement: Supplementary file 4 — Supporting information. [file PCE-45-1474-s003.jpg]

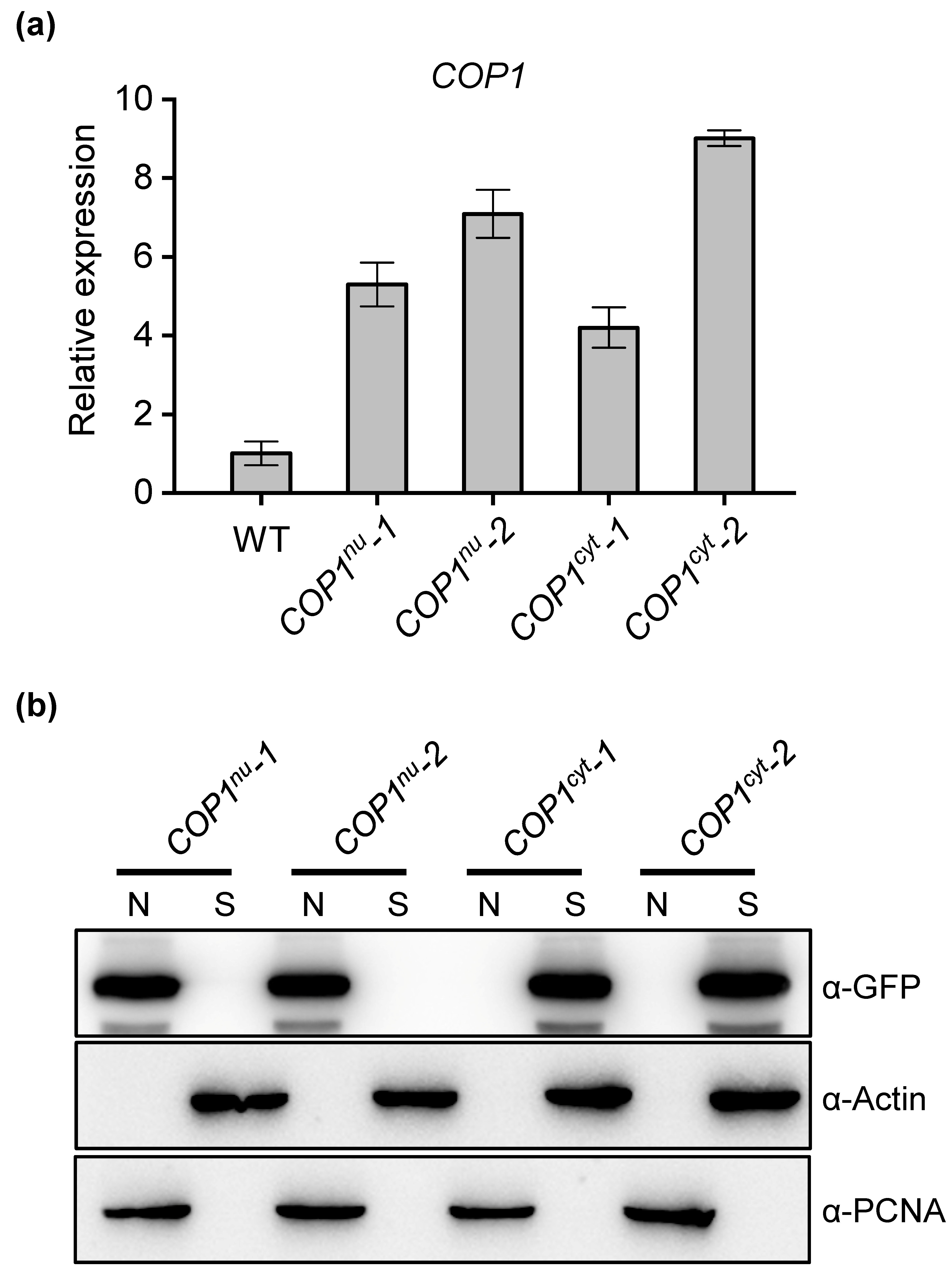

Supplement: Supplementary file 5 — Supporting information. [file PCE-45-1474-s002.jpg]

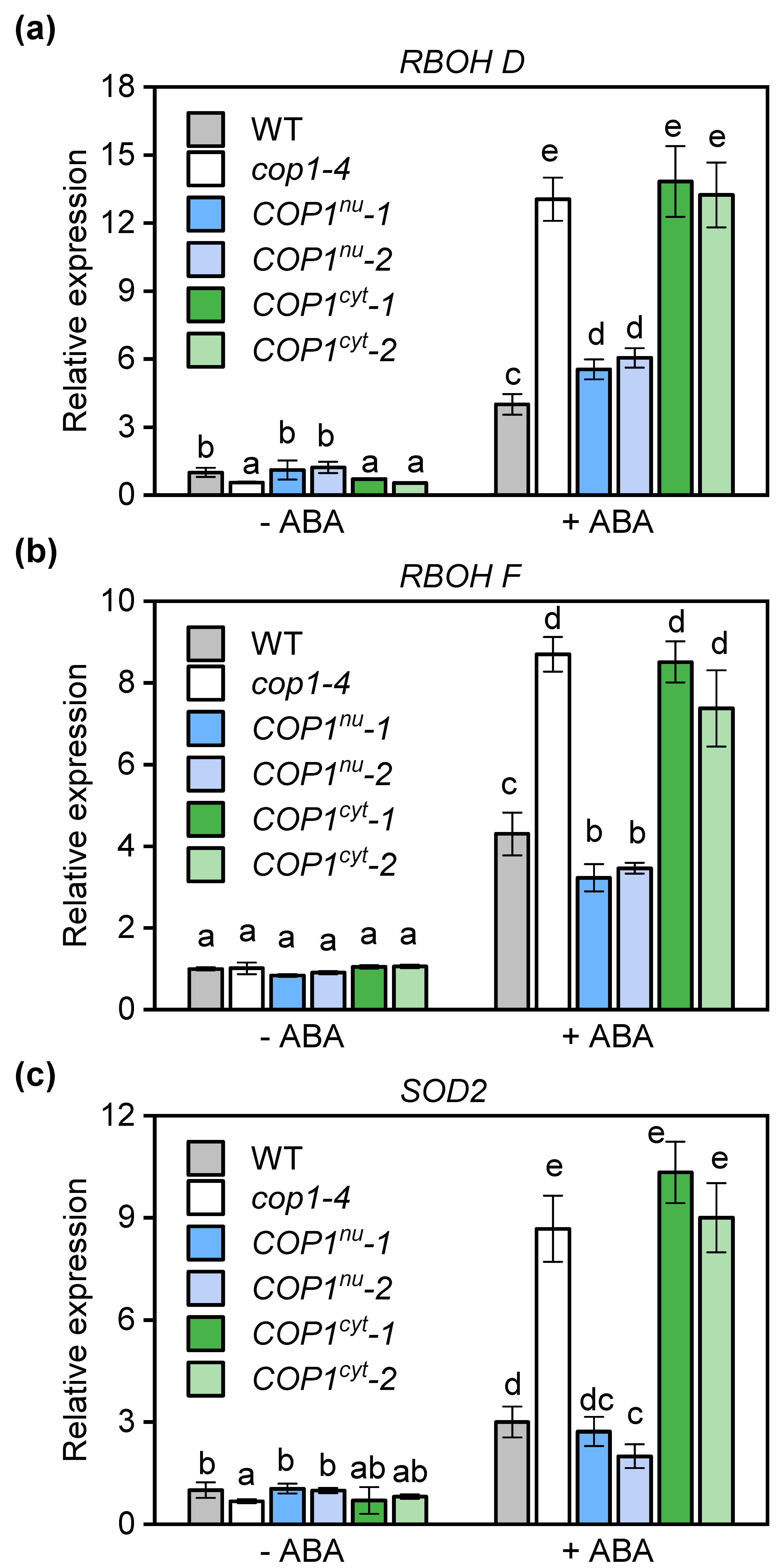

Supplement: Supplementary file 6 — Supporting information. [file PCE-45-1474-s004.jpg]

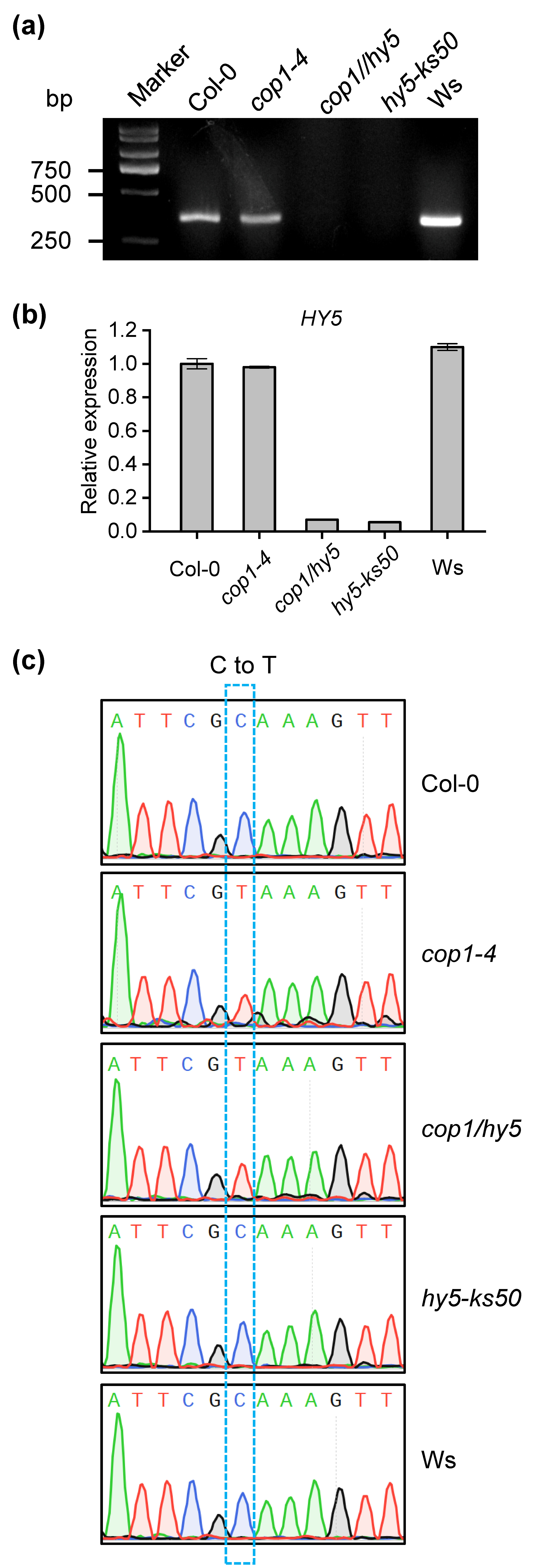

Supplement: Supplementary file 7 — Supporting information. [file PCE-45-1474-s001.jpg]

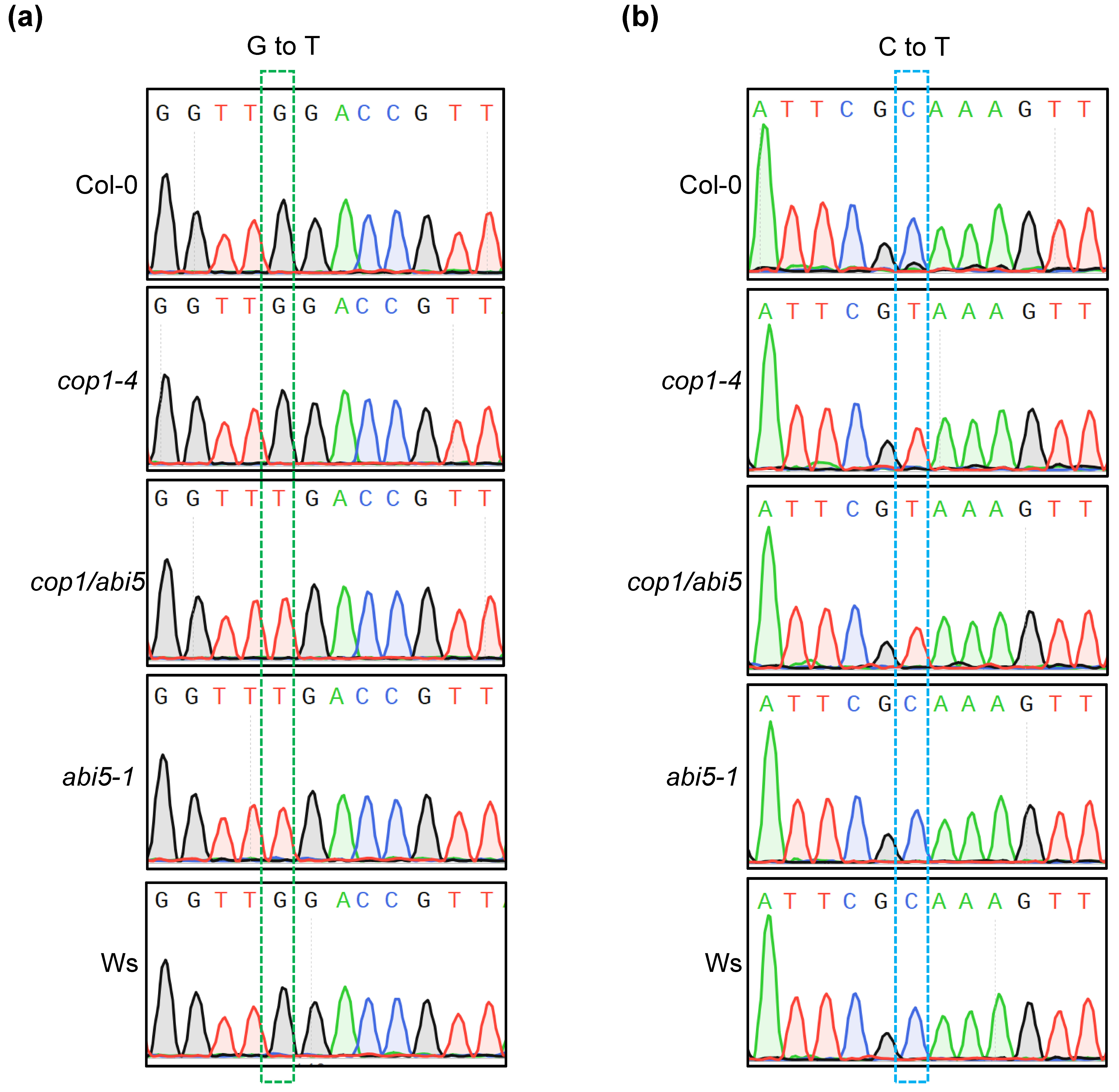

Supplement: Supplementary file 8 — Supporting information. [file PCE-45-1474-s007.jpg]

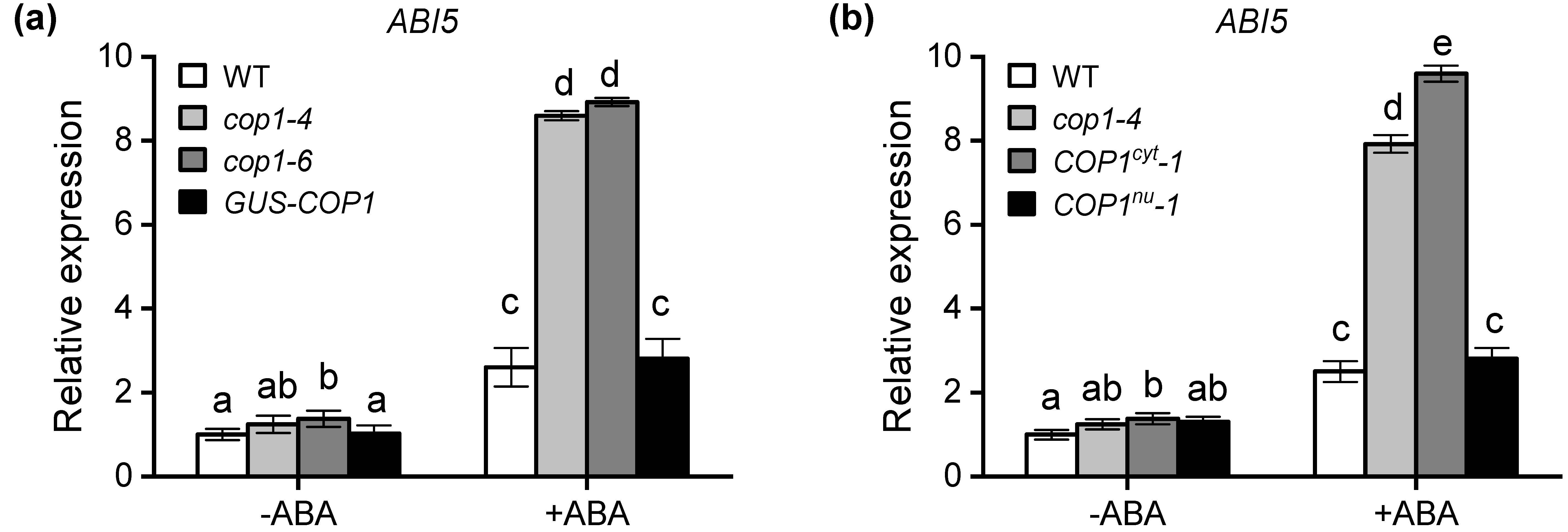

Supplement: Supplementary file 9 — Supporting information. [file PCE-45-1474-s006.jpg]

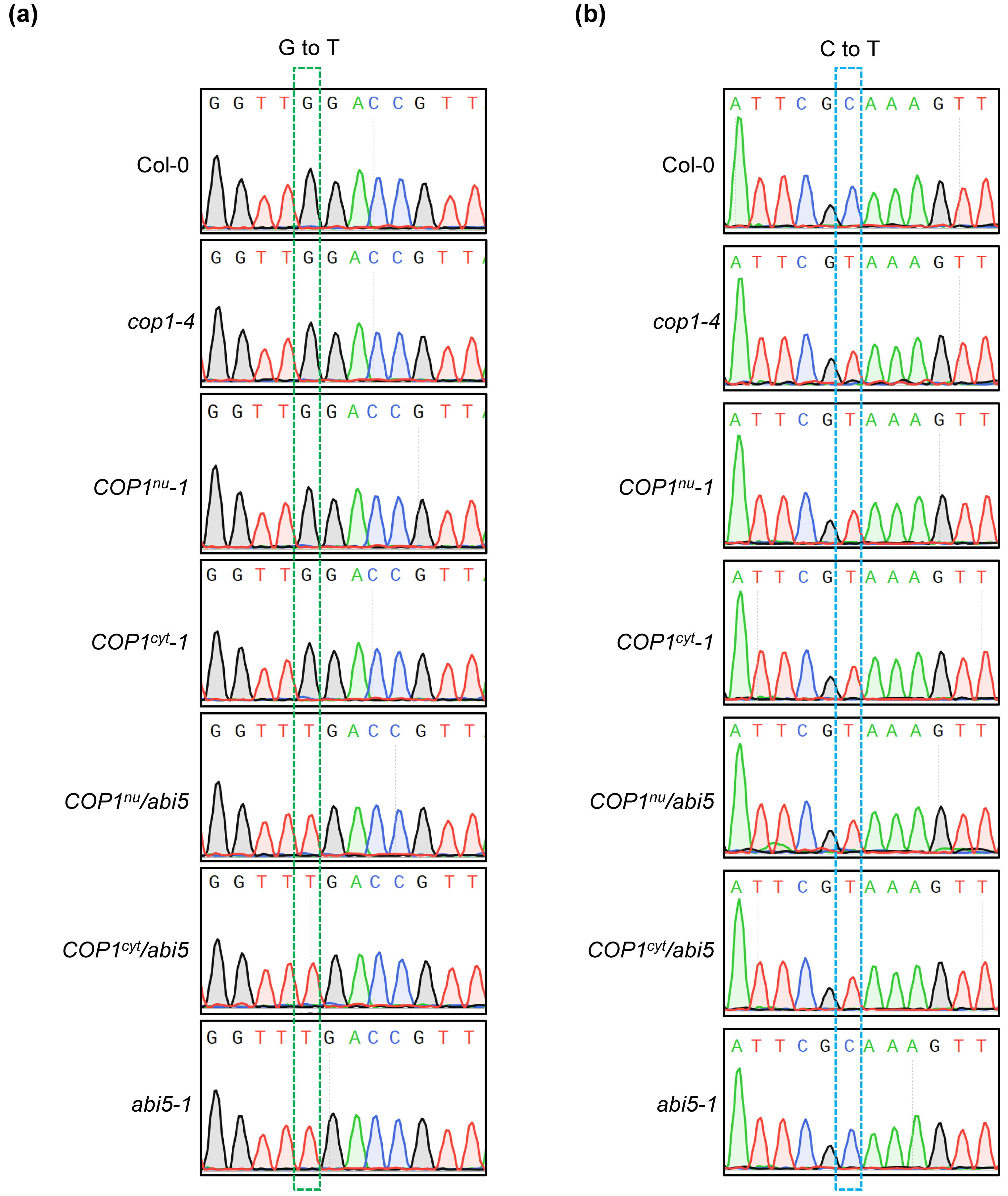

Supplement: Supplementary file 10 — Supporting information. [file PCE-45-1474-s009.jpg]
