# Supplementary material for: Abscisic acid‐induced cytoplasmic translocation of constitutive photomorphogenic 1 enhances reactive oxygen species accumulation through the HY5‐ABI5 pathway to modulate seed germination
Source: Plant Cell Environ. 2022 Mar 10;45(5):1474–89. doi: 10.1111/pce.14298 (PMC9311139; doi:10.1111/pce.14298)
Supplement: Supplementary file 11 — Supporting information. [file PCE-45-1474-s010.docx]

**SUPPORTING INFORMATION**

**Title:** ABA-induced cytoplasmic translocation of COP1 enhances ROS accumulation through the HY5-ABI5 pathway to modulate seed germination

**Authors:** Qing-Bin Chen^a1^, Wen-Jing Wang^b1^, Yue Zhang^a^, Qi-Di Zhan^a^, Kang Liu^a^, José Ramón Botella^c^, Ling Bai^a^*, and Chun-Peng Song^a^*

**Affiliation:**

^a^ State Key Laboratory of Crop Stress Adaptation and Improvement, School of Life Sciences, Henan University, Kaifeng 475001, China

^b^ Department of Biology and Food Science, Shangqiu Normal University, Shangqiu 476000, China.

^c^ Plant Genetic Engineering Laboratory, School of Agriculture and Food Sciences, The University of Queensland, Brisbane, Queensland 4072, Australia

^1^These authors contributed equally to this article.

***Correspondence**: Ling Bai

**Tel:** +86 136 5378 2901

**Email:** bailing@henu.edu.cn

***Correspondence**: Chun-Peng Song

**Tel:** +86 137 0378 1918

**Email:** songcp@henu.edu.cn

Qing-Bin Chen ORCID <https://orcid.org/0000-0003-2900-5852>

Wen-Jing Wang ORCID <https://orcid.org/0000-0002-4513-2280>

José Ramón Botella ORCID <https://orcid.org/0000-0002-4446-3432>

Ling Bai ORCID <https://orcid.org/0000-0001-9105-0377>

Chun-Peng Song ORCID <https://orcid.org/0000-0001-8774-4309>

**Running head:** COP1 modulates ABA-mediated inhibition of seed germination

**SUMMARY STATEMENT**

COP1, the core repressor of light signals, functions in ABA-mediated inhibition of *Arabidopsis* seed germination. The ABA-induced COP1 cytoplasmic translocation increases both HY5 and ABI5 protein levels in the nucleus, leading to elevated expression of ABI5 target genes and enhanced ROS levels.

**Supplementary Information Text**

**Supplementary materials and Methods**

**CFP-COP1 subcellular localization**

To observe the effects of ABA and/or GSH on COP1 localization, the seeds of *CFP-COP1* transgenic material were germinated on MS medium contained 0 μM ABA plus 0 μM GSH, or 0.5 μM ABA, or 0.5 μM ABA plus 300 μM GSH, or 300 μM GSH for 48 h, and the fluorescence of CFP-COP1 in the seeds was observed. The cover slides were gently tapped with tweezers to disperse the seeds into single cells, and the Zeiss LSM 710 scan confocal microscope was used to observe and take photos. CFP Fluorescence was visualized at an excitation wavelength of 405 nm and emission wavelength of 470 to 500 nm. DIC (Differential-Interference-Contrast) channel is used to observe the cell structure in the bright field.

**GFP-COP1^cyt^ and GFP-COP1^nu^ Subcellular Localization**

To visualize GFP-COP1^cyt^ and GFP-COP^nu^ subcellular localization, the combinations of GFP-COP1^cyt^ or GFP-COP^nu^ with H2B-mCherry were co-transformed into *Arabidopsis* protoplasts. H2B-mCherry was expressed as a marker protein for nuclear localization. The *Arabidopsis* protoplasts were then cultured for 16 h, and the fluorescence images were captured by using a Zeiss LSM 710 scan confocal microscope.

**DATA AVAILABILITY STATEMENT**

Data available on request from the authors

**Supplementary Figure 1. *cop1* mutants show hypersensitivity to ABA during germination.**

Germination rates of WT, *cop1-4*, *cop1-6* and *GUS-COP1* seeds germinated in the absence or presence of 0.1 μM ABA. After vernalization, plates were placed in an incubator and recorded as 0 h. Germination was measured every 12 h until 96 h. Data are means ± SD, (*n* > 50 seeds per genotype) of three replicates.

**Supplementary Figure 2. Germination phenotypes of WT, *cop1-4*, *cop1-6* and *GUS-COP1* seeds on the medium containing ABA and/or GSH.**

WT, *cop1-4*, *cop1-6* and *GUS-COP1* seeds germinated on MS medium supplemented with different amounts of GSH and/or ABA for 48 h. (Scale = 0.5 mm)

**Supplementary Figure 3. Subcellular localization of CFP-COP1 in the presence of ABA and/or GSH.**

(a) *CFP-COP1* seeds were treated as described in (Figure S2). The concentrations of ABA and GSH were 0.5 μM and 300 μM, respectively. The CFP channel shows the location of CFP-COP1 within single cells. DIC (Differential-Interference-Contrast) channels display the structure of single cells in bright field.

(b) The number of cells showing nuclear enriched CFP-COP1 in (a) was counted. Data are means ± SD, *n* = about 50 cells for three independent experiments. Different letters indicate statistical differences at *P* < 0.05 (one-way ANOVA analysis).

(c) O_2_^-^ concentration was measured for the material shown in (a). Data are means ± SD of three independent experiments. Different letters indicate statistical differences at *P* < 0.05 (one-way ANOVA analysis).

(d) H_2_O_2_ concentration was measured for the material shown in (a). Data are means ± SD of three independent experiments. Different letters indicate statistical differences at *P* < 0.05 (one-way ANOVA analysis).

**Supplementary Figure 4. Subcellular localization of COP1^nu^ and COP1^cyt^.**

GFP-COP1^nu^ and GFP-COP1^cyt^ were expressed in *Arabidopsis* protoplasts using a transient transformation system, and incubated in a buffer containing 0 μM or 10 μM ABA for 3 h, before being observed using confocal microscopy. Green fluorescence indicates the location of GFP-COP1^nu^ and GFP-COP1^cyt^. Blue fluorescence (false color) represents chloroplast spontaneous fluorescence. The red fluorescence indicates the location of H2B-mcherry, which was used as a nuclear localization marker (Scale bar: 5 μM).

**Supplementary Figure 5. Characterization of COP1 nuclear localization mutants *COP1^nu^* and COP1 cytoplasmic localization mutants *COP1^cyt^*.**

(a) *COP1* relative expression levels in *COP1^nu^* and *COP1^cyt^* lines measured by qRT-PCR. The values are means ± SD of three replicates.

(b) Localization of COP1 protein in *COP1^nu^* and *COP1^cyt^* lines. Nuclear and cytoplasmic protein fractions were obtained for *COP1^nu^-1*, *COP1^nu^-2*, *COP1^cyt^-1* and *COP1^cyt^-2*, and COP1 protein levels detected using an anti-GFP antibody. ACTIN was used as an internal reference for cytoplasmic proteins while PCNA was used as an internal reference for nuclear proteins.

**Supplementary Figure 6. Cytoplasm localized COP1 enhances *RBOH D*, *RBOH F* and *SOD2* expression levels in response to ABA during seed germination.**

Relative transcription levels of *RBOH D*, *RBOH F* and *SOD2* in WT, *cop1-4*, *COP1^nu^-1*, *COP1^nu^-2*, *COP1^cyt^-1* and *COP1^cyt^-2* seeds germinated for 36 h on medium containing 0 μM or 10 μM ABA quantified by qRT-PCR. Data are means ± SD of three replicates. Different letters indicate statistical differences at *P* < 0.05 (one-way ANOVA analysis).

**Supplementary Figure 7. Identification of *cop1-4/hy5-ks50* double homozygous mutants.**

(a) Identification of T-DNA insertion in *hy5-ks50*. *hy5-ks50* is a T-DNA insertion mutant. PCR with primers flanking the T-DNA insertion were used to amplify genomic DNA. Lanes lacking an amplicon identify homozygous *hy5-ks50* mutations.

(b) Relative *HY5* expression levels were detected in Col-0, *cop1-4*, *cop1-4/hy5-ks50*, *hy5-ks50* and Ws by qRT-PCR. Data are means ± SD of three replicates.

(c) Sequencing of genomic amplicons to determine *cop1-4* mutation zygosity. Genomic fragments were amplified using primers proximal to the mutation site in *cop1-4* and the amplicons sequenced.

**Supplementary Figure 8. Identification of *cop1-4/abi5-1* double homozygous mutants.**

(a) Identification of *abi5-1* homozygosity in *cop1-4/abi5-1* double mutants was determined by sequencing. Genomic fragments were amplified using primers proximal to the mutation site in *abi5-1* and the amplicons sequenced.

(b) Identification of *cop1-4* homozygosity in *cop1-4/abi5-1* double mutants was determined by sequencing. Genomic fragments were amplified using primers proximal to the mutation site in *cop1-4* and the amplicons sequenced.

**Supplementary Figure 9. COP1 is involved in the regulation of ABA-induced *ABI5* expression.**

(a) Relative *ABI5* transcription levels in WT, *cop1-4*, *cop1-6*, and *GUS-COP1* seeds were quantified by qRT-PCR. Seeds were germinated on medium supplemented with 0 μM or 0.5 μM ABA for 36 h. Data are means ± SD of three replicates. Different letters indicate statistical differences at *P* < 0.05 (one-way ANOVA analysis).

(b) Relative *ABI5* transcription levels in WT, *cop1-4*, *COP1^cyt^-1*, *COP1^nu^-1* seeds were quantified by qRT-PCR. Seeds were germinated on medium supplemented with 0 μM or 0.5 μM ABA medium for 36 h. Data are means ± SD of three replicates. Different letters indicate statistical differences at *P* < 0.05 (one-way ANOVA analysis).

**Supplementary Figure 10.** **Identification of *COP1^nu^-1/abi5-1* and *COP1^cyt^-1/abi5-1* double homozygous mutants**.

(a) The zygosity for the *abi5-1* mutation in *COP1^nu^-1/abi5-1* and *COP1^cyt^-1/abi5-1* double mutant was determined by sequencing. Primers proximal to the *abi5-1* mutation were used to amplify genomic DNA and the amplicon sequenced.

(b) The zygosity for the *cop1-4* mutation in *COP1^nu^-1/abi5-1* and *COP1^cyt^-1/abi5-1* double mutant was determined by sequencing. Primers proximal to the *cop1-4* mutation were used to amplify genomic DNA and the amplicon sequenced.

Supplementary Table 1. Primer sequences used in the manuscript.

| Primer | Nucleotide sequence |
| --- | --- |
| *cop1-4*-geno-F | 5' CCGTTGAGAGACATAGAA 3' |
| *cop1-4*-geno-R | 5' TGACCAAGAGCTGAGTAG 3' |
| *hy5-ks50*-geno-F | 5' ATCAAGCAGCGAGAGGTC 3' |
| *hy5-ks50*-geno-R | 5' AAGTTTCTTTTCCGACAG 3' |
| *abi5-1*-geno-F | 5' GTGATCCTTCATTCCCGG 3' |
| *abi5-1*-geno-R | 5' CTCTTTTCCTTCCCCTTA 3' |
| qRT-COP1-F | 5' AAACTTCAGCTCGGCATGTGT 3' |
| qRT-COP1-R | 5' CTTTGTAGTGCTTCCCGAAACTG 3' |
| qRT-HY5-F | 5' CCATCAAGCAGCGAGAGGTCATCAA 3' |
| qRT-HY5-R | 5' CGCCGATCCAGATTCTCTACCGGAA 3' |
| qRT-ABI5-F | 5' TGGGTGACCCATCAGGTTATGC 3' |
| qRT-ABI5-R | 5' TCTGAAGACACCGGGCTTAAC 3' |
| qRT-EM1-F | 5' CGAGGAAGGAGCAGTTAG 3' |
| qRT-EM1-R | 5' GTACTGAGTCCTCCTTTACG 3' |
| qRT-EM6-F | 5' TGAGAGGGCAAAGAAGGG 3' |
| qRT-EM6-R | 5' CTTGTCTCCGGTGCTAAG 3' |
| qRT-RD29A-F | 5' GACGAGCTAGAACCTGAAGTG 3' |
| qRT-RD29A-R | 5' TGCATCGTGTCCGTAAGAG 3' |
| qRT-RAB18-F | 5' GAAGAACATGGCGTCTTACC 3' |
| qRT-RAB18-R | 5' CAGTTCCAAAGCCTTCAGTC 3' |
| qRT-RBOH D-F | 5' GGATTCAAATACAAAAGTGGACAG 3' |
| qRT-RBOH D-R | 5' GGATATGTACGCTCAGGTAATCG 3' |
| qRT-RBOH F-F | 5' ACATTGTCTCTGGCACTAGGGT 3' |
| qRT-RBOH F-R | 5' TACTCCGCAATAAAACACTCCTA 3' |
| qRT-SOD2-F | 5' CTCATTCCTCCTTCCTCCAATC 3' |
| qRT-SOD2-R | 5' GCTTTAACGGCGAAGGAAAC 3' |
| qRT-Actin2-F | 5' ATTACCCGATGGGCAAGTCA 3' |
| qRT-Actin2-R | 5' CACAAACGAGGGCTGGAACA 3' |
